# Comparative analysis on rhizosphere microorganisms of *Cynodon dactylon* (Poaceae) from the natural restoration area and eco-restoration engineering area in water-level-fluctuation zone of Jinsha River-type reservoirs, China

**DOI:** 10.3897/BDJ.13.e159524

**Published:** 2025-12-30

**Authors:** Lanfang Zhou, Shengjun Wu, Maohua Ma

**Affiliations:** 1 School of River and Ocean Engineering, Chongqing Jiaotong University, Chongqing, China School of River and Ocean Engineering, Chongqing Jiaotong University Chongqing China; 2 Key Laboratory of Reservoir Aquatic Environment, Chongqing Institute of Green and Intelligent Technology, Chinese Academy of Sciences, Chongqing, China Key Laboratory of Reservoir Aquatic Environment, Chongqing Institute of Green and Intelligent Technology, Chinese Academy of Sciences Chongqing China; 3 Chongqing School, University of Chinese Academy of Sciences, Chongqing, China Chongqing School, University of Chinese Academy of Sciences Chongqing China

**Keywords:** water-level-fluctuation zone, rhizosphere microorganism, biodiversity, eco-restoration engineering

## Abstract

Rhizosphere microorganisms may play an important role in plant growth and environmental adaptation during eco-restoration engineering in the water-level-fluctuation zone (WLFZ) of large river-type reservoirs. The purpose of this study is to compare the rhizosphere microbial community features of *Cynodon dactylon* derived from the natural restoration area (NEE) and eco-restoration engineering area (YEE) in the WLFZ of Jinsha River-type reservoirs using high-throughput sequencing and bioinformatic analyses. The dominant taxa were Proteobacteria and Ascomycota in *C. dactylon* rhizosphere from both NEE and YEE. The eco-restoration engineering improved the α-diversity of bacterial community, but reduced the diversity of fungal community. Eco-restoration engineering did not change the dominant pattern of aerobic respiration I (cytochrome c) [PWY-3781] and aerobic respiration II (cytochrome c, yeast) [PWY-7279] metabolic pathways in the rhizosphere microbial community. Many microbes (such as Alphaproteobacteria and Sphingomonadaceae) in YEE were identified as significantly enriched biomarkers, while the key biomarkers in the rhizosphere in NEE are relatively limited where Burkholderiaceae and Burkholderiales may be the key characteristic microbe. Our study provides a theoretical basis and potential clue for understanding the role of eco-restoration engineering in the re-assembly of rhizosphere microorganisms and supporting vegetation restoration in the WLFZ of large river-type reservoirs.

## Introduction

The water-level-fluctuation zone (WLFZ) in the river-type reservoir of Jinsha River Basin is a key area of ecological protection in southwest China. River-type reservoirs are narrow waterbodies formed by intercepting natural rivers, extending along the original riverbed with dual characteristics of lake and river (Wu et al. 2022). Due to the development of cascade hydropowers, a large area of WLFZ has been formed in this region, which is faced with vegetation degradation, soil erosion and biodiversity reduction. As an important section of Jinsha River ecosystem, the WLFZ’s plants play a key role in maintaining its structural stability (Jiang et al. 2022). Plant’s rhizosphere is an essential region for plant health, which enriches various microbes to improve soil nutrient availability (Liu et al. 2024). Rhizosphere microbes have been reported to be important to the survival of plants in the WLFZ of large reservoirs (Zhou et al. 2023), while plant properties (e.g. sex and type) also can alter rhizosphere microorganism assembly of plants (Lan et al. 2025).

With continuous operating of hydropower stations, the structure and function of rhizosphere microorganisms in plants of WLFZ face strong waterlogging stress (Zhou et al. 2024). Abiotic stresses may change the metabolic processes or pathways of rhizosphere microorganisms (Li et al. 2024). This may lead to the shift of self-protection to auto-toxicity in the plant’s rhizosphere, which will deteriorate the plant health (Li et al. 2025). Eco-restoration is believed to be helpful in alleviating the imbalance and maintaining soil ecological health. It has been demonstrated that, during eco-restoration, plants are able to adapt to the restoration processes through non-randomly assembling of rhizosphere microbial communities and metabolic functions in abandoned lead-zinc mines (Gao et al. 2025). Up to now, the microbial community structure and metabolic pathway during eco-restoration processes in the WLFZ of Jinsha River-type reservoir remain unclear.

In recent years, several eco-restoration projects have been performed in the WLFZ of large river-type reservoirs in the Jinsha River Basin (Jin et al. 2025). Based on the eco-restoration engineering carried out in WLFZ of Wudongde and Baihetan Reservoirs, the appropriate plant configuration and engineering technology were put forward to improve the ecological control effect of the WLFZ and the restoration mode was very important for the restoration of the WLFZ by comprehensively considering the plant ecological characteristics and external factors (Jin et al. 2025). The investigation of plant community structure and species diversity in the experimental area of eco-restoration in the WLFZ of Wudongde Reservoir showed that the experimental area was rich in plant species, mainly Compositae, Gramineae and Leguminosae; after flooding, the plants adapted to land may gradually disappear and the plants tolerant to flooding may increase (Wan et al. 2022).

In this study, *Cynodon dactylon*, a typical dominant plant in the reservoir WLFZ of Jinsha River Basin, was taken as a representative. In addition, *C. dactylon* is the common dominant species in the WLFZ of many reservoirs, which can reproduce asexually, based on its axillary buds of stolon and rhizome nodes (Yi et al. 2024). The rhizosphere soil of WLFZ derived from the natural restoration area (NEE) at QianWanguan Township, Leibo County, Liangshan Yi Autonomous Prefecture, Sichuan Province and the eco-restoration engineering area (YEE) at Xinmin Village, Wuding County, Chuxiong Yi Autonomous Prefecture, Yunnan Province was collected in the adjacent period, respectively. The similarities and differences of the structure, diversity and metabolic function of rhizosphere microbial community in these two types of areas were compared and analysed. The key biomarkers showing significant differences between NEE and YEE were identified, providing a theoretical basis and potential practical method for understanding the role of eco-restoration engineering in the evolution of rhizosphere microorganisms and supporting vegetation restoration in the WLFZ of large river-type reservoirs.

## Materials and methods

**Natural restoration area and eco-restoration engineering area**

**Figure 1.**
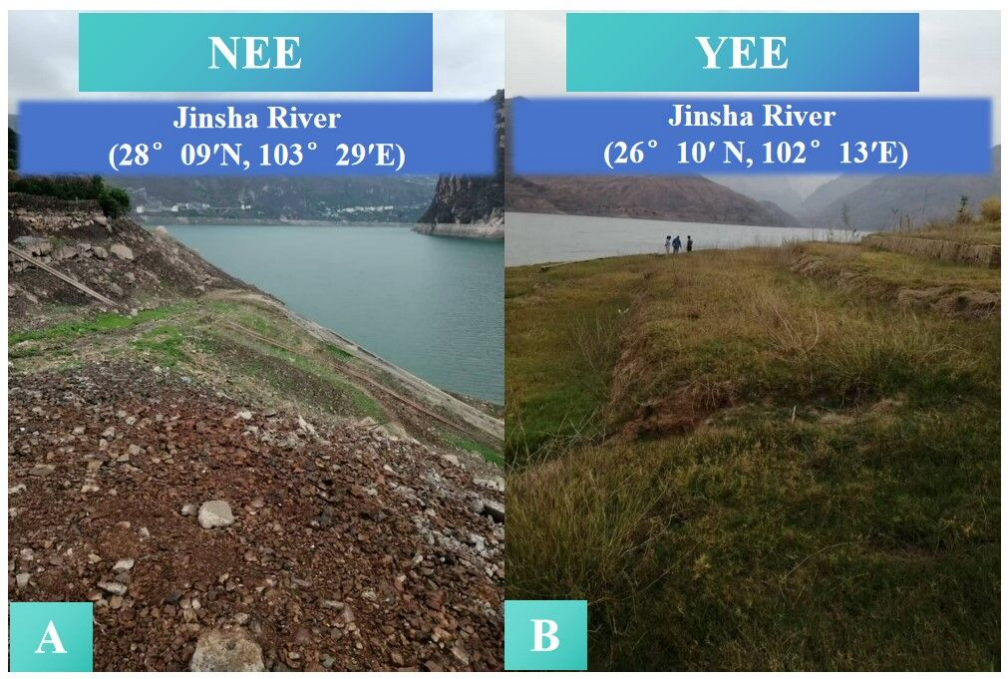
*Cynodon dactylon* from the WLFZ of NEE (A) and YEE (B) in the Jinsha River Reservoir.

A scientific investigation on the WLFZ of Jinsha River mainstream reservoirs was jointly organised by researchers from Chongqing Institute of Green Intelligent and Technology, Chinese Academy of Sciences and Chongqing Branch of Changjiang River Scientific Research Institute in 2022. Affected by the dry hot valley climate and the disturbance of hydropower projects, vegetation showed a sparse patchy distribution pattern. Through field observation, plant quadrats and literature research, the present study finally selected the Jinsha River WLFZ 1 (28°09′N，103°29′E) and WLFZ 2 (26°10′N, 102°13′E) as a natural restoration area (NEE) and an eco-restoration engineering area (YEE), respectively. These both areas are near QianWanguan Township, Leibo County, Liangshan Yi Autonomous Prefecture, Sichuan Province and the eco-restoration zone of WLFZ at Xinmin Village, Wuding County, Chuxiong Yi Autonomous Prefecture, Yunnan Province, respectively (Fig. 1). The paper recently published by Chongqing Branch of Changjiang River Scientific Research Institute gives a detailed introduction to this YEE (Jin et al. 2025). Average annual temperature in the Jinsha River Basin is reported to be 10.2°C, average annual precipitation is 1000 mm in the lower reaches and about 152 billion m^3^ of mean annual runoff is recorded (Yin et al. 2018).

**Soil sampling**

Rhizosphere soil is the layer immediately surrounding plant roots. Rhizosphere soil of *C. dactylon* from NEE and YEE was sampled from the above study areas by shaking roots and making the soil into the sterile tubes. The collected rhizosphere soil was immediately placed in a -20℃ car refrigerator for short-term storage and was transferred into a -80℃ freezer after returning to the laboratory. For each of NEE and YEE, rhizosphere soil from 10-15 individuals was collected in 5-8 sites. Then soil samples were mixed manually and were evenly divided into three parts which were put into three sterile tubes, in order to decrease the impact of spatial heterogeneity of samples, improve the representativeness of samples and the reliability of detection results, as well as meeting the soil amount required for sequencing (Li et al. 2016, Xu et al. 2020, Malviya et al. 2022). In addition, variation of microbial community in-between samples at the phylum, class, order, family and genus levels were also investigated, as shown in Suppl. material 1 (Figs. S1-S10).

**Soil DNA extraction and Illumina NovaSeq sequencing**

Rhizosphere soil DNA from NEE and YEE were extracted separately using CTAB method (Griffiths et al. 2000). Primers 341F (5′-CCTAYGGGRBGCASCAG-3′) and 806R (5′-GGACTACNNGGGTATCTAAT-3′) were adopted to amplify the V3-V4 region of 16s rDNA (Berg et al. 2012, Michelsen et al. 2013). Meanwhile, primers ITS1F (5′-CTTGGTCATTTAGAGGAAGTAA-3′) and ITS2R (5′-GCTGCGTTCTTCATCGATGC-3′) were used to amplify fungal ITS region (Li et al. 2016, Xiong et al. 2017).

Phusion® High-Fidelity PCR Master Mix with 10 ng template DNA and forward and reverse primers were used for PCR reactions which were performed with initial denaturation (98℃, 1 min), 30 cycles of denaturation (98℃, 10 s), annealing (50℃, 30 s), elongation (72℃, 30 s) and then final extension (72℃, 5 min).

NEB Next® Ultra DNA Library Prep Kit (Illumina, USA) was selected to produce sequencing libraries. After finishing the quality assessment, high-throughput sequencing was carried out on Illumina NovaSeq platform. Raw data have been deposited into the NCBI Bioproject repository with an ID of SUB15472619.

**Data analyses**

Raw sequence data were treated using QIIME2 software (Bolyen et al. 2019) where qiime tools import the programme for format transformation, dada2 plugin for quality control, de-noising and mergence, feature-classifier plugin for producing the taxonomy table and feature-table plugin for removing the contaminated mitochondrial and chloroplast sequences were used. The database used to assign the sequences are UNITE and GREEGENES. Venn plots were used to show unique and common ASVs between different samples. Alpha diversity indices including Observed species richness (observed_features), Chao1 estimator of species richness (Chao1 index), Shannon-Wiener diversity index (Shannon index), Simpson's diversity index (Simpson index) and Faith's phylogenetic diversity index (Faith_pd index) were calculated to assess the microbial diversity following a previous study (Zhou et al. 2024). The key biomarkers were identified using linear-discriminant-analysis effect size (LEfSe) with a logarithmic LDA score of no less than 4 (Chang et al. 2022). The prediction of metabolic pathways was carried out using PICRUSt2 softwar (Douglas et al. 2020) and the statistical of microbial function refers to the KEGG level pathway and MetaCyc pathway (Caspi et al. 2020). PICRUSt software has been widely used to explore the function of rhizosphere microorganisms of *Miscanthus* (Chen et al. 2020) Gannan Navel Orange (Zhou et al. 2021), *Glycyrrhiza uralensis* Fisch (Dong et al. 2021), *Cucurbita pepo* L. (Wang et al. 2022) and *Suaeda salsa* (Yu et al. 2022). The prediction of bacterial ecological function, based on 16S rRNA amplicon data, is completed by FAPROTAX (v.1.2.6) (Sansupa et al. 2021). ASV classification information was imported into FAPROTAX to realise ecological function mapping. Fungal functional groups were predicted using FUNGuild, a tool that assigns ecological roles, based on fungal taxonomy, phylogenetic relationships and a curated database of functional traits and fungi OTU/ASV were classified into saprophytic, pathogenic, symbiotic and other functional groups (Nguyen et al. 2016). Network analysis was adopted to find the key microbes that are linked to each other using Wekemo Bioincloud (Gao et al. 2024) which analyses Spearman’s rank correlations between predominant taxa. The above analyses were carried out by referring the "Atacama soil microbiome tutorial" together with the customised programme scripts (https://docs.qiime2.org/2019.1/) and Bioincloud (https://www.bioincloud.tech/) on 1 March 2025. Unless specified above, the parameters used in the normalisation procedures and statistical methods were set as default.

## Results and discussion

**Rhizosphere microbial community structure in NEE and YEE**

**Figure 2.**
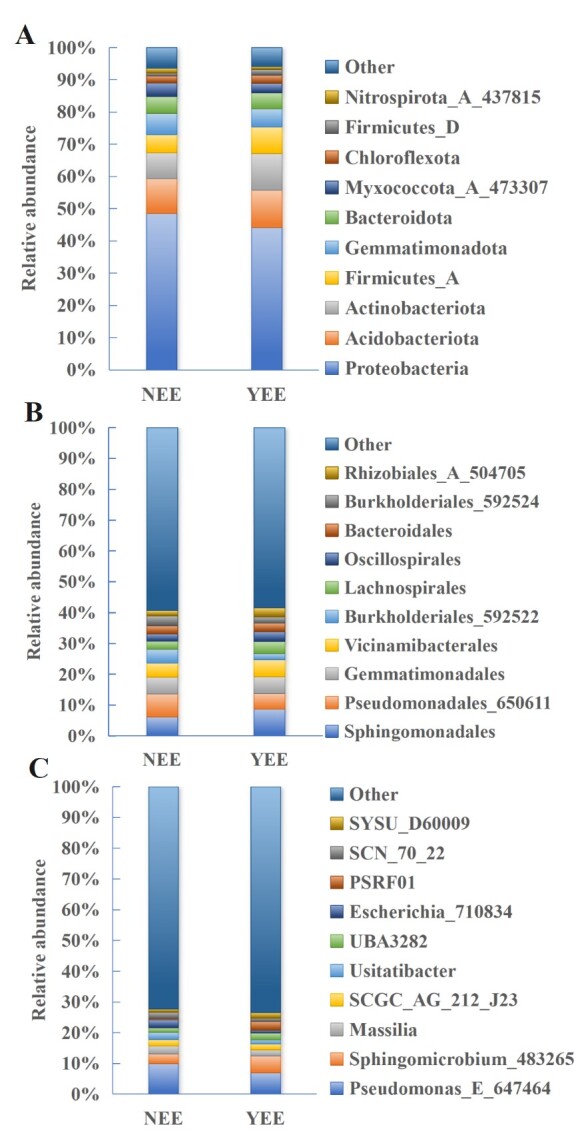
Composition of rhizosphere bacteria in C. dactylon of NEE and YEE at the phylum (A), order (B) and genus (C) levels. SYSU_D60009 belongs to Dongiaceae; SCN_70_22 belongs to Gemmatimonadaceae; PSRF01 belongs to Pyrinomonadaceae; UBA3282 belongs to Lachnospiraceae; SCGC_AG_212_J23 belongs to Gammaproteobacteria. The relative abundance of various bacteria in NEE was the average of NEE -1, NEE -2 and NEE -3; the relative abundance of various bacteria in YEE is the average of YEE -1, YEE -2 and YEE-3.

Affected by waterlogging stress or climatic conditions, the vegetation in the WLFZ of large river-type cascade reservoirs presents problems, such as single species and vegetation degradation. In order to alleviate this problem, some eco-restoration projects have been implemented in these regions (Jin et al. 2025). In the present study, a typical YEE was selected as above to carry out the comparative analysis of rhizosphere microorganisms with the NEE. In the rhizosphere bacterial communities of *C. dactylon* located in NEE and YEE, the relative abundance of Proteobacteria is the highest at the phylum level, accounting for 48.5% and 44.1%, respectively (Fig. 2A). Proteobacteria is one of the largest branches in bacterial domain (Sharma et al. 2022). Additionally, Proteobacteria was often observed to be most abundant in the soil, characterised by high diversity in morphology, physiology and metabolic functions (Spain et al. 2009). However, there are differences in the dominant phyla after Proteobacteria. The dominant phyla in NEE was Acidobacteria (10.8%), Actinobacteria (8.1%) and Firmicutes (5.6%) and the dominant phyla following the most dominant phyla in YEE was the same as NEE, but the abundance changed, namely Acidobacteria (11.5%), Actinobacteria (11.4%) and Firmicutes (8.3%). The relative abundance of the dominant phylum Proteobacteria in the rhizosphere soil of *C. dactylon* in NEE is 4.4% higher than in the YEE region. At the class level (Suppl. material 1 Fig. S11), the most abundant classes are Alphaproteobacteria (23.4%) and Gammaproteobacteria (20.9%) in YEE. These two classes are also most abundant in NEE, being 16.5% and 32.0%, respectively. At the order level, the bacterial community structures of NEE and YEE differ greatly in the relative abundance of dominant bacteria (Fig. 2B). The most dominant bacterial orders were found to be Sphingomonadales (8.7%), Gemmatimonadales (5.5%), Vicinamibacterales (5.4%) and Pseudomonadales_650611 (5.0%), while the most dominant bacterial orders in NEE were Pseudomonadales_650611 (7.6%), Sphingomonadales (6.1%) and Gemmatimonadales (5.4%). It was found that Sphingomonadales was also dominant in the rhizosphere soil of six plants (*Artemisia argyi*, *Erigeron annuus*, *Ageratum conyzoides*, *Euphorbia hirta*, *Bidens biternata* and *Viola japonica*) (Lei et al. 2018). At the family level (Suppl. material 1, Fig. S12), the most dominant family is different between NEE and YEE, which is Pseudomonadaceae for NEE and Sphingomonadaceae for YEE, respectively. At the genus level, the dominant genera of NEE and YEE are *Pseudomonas_E_647464* (Fig. 2C), but it is less abundant in YEE. The relative abundance of *Pseudomonas_E_647464* (7.0%) and *Sphingomicrobium_483265* (5.5%) is very close.

**Figure 3.**
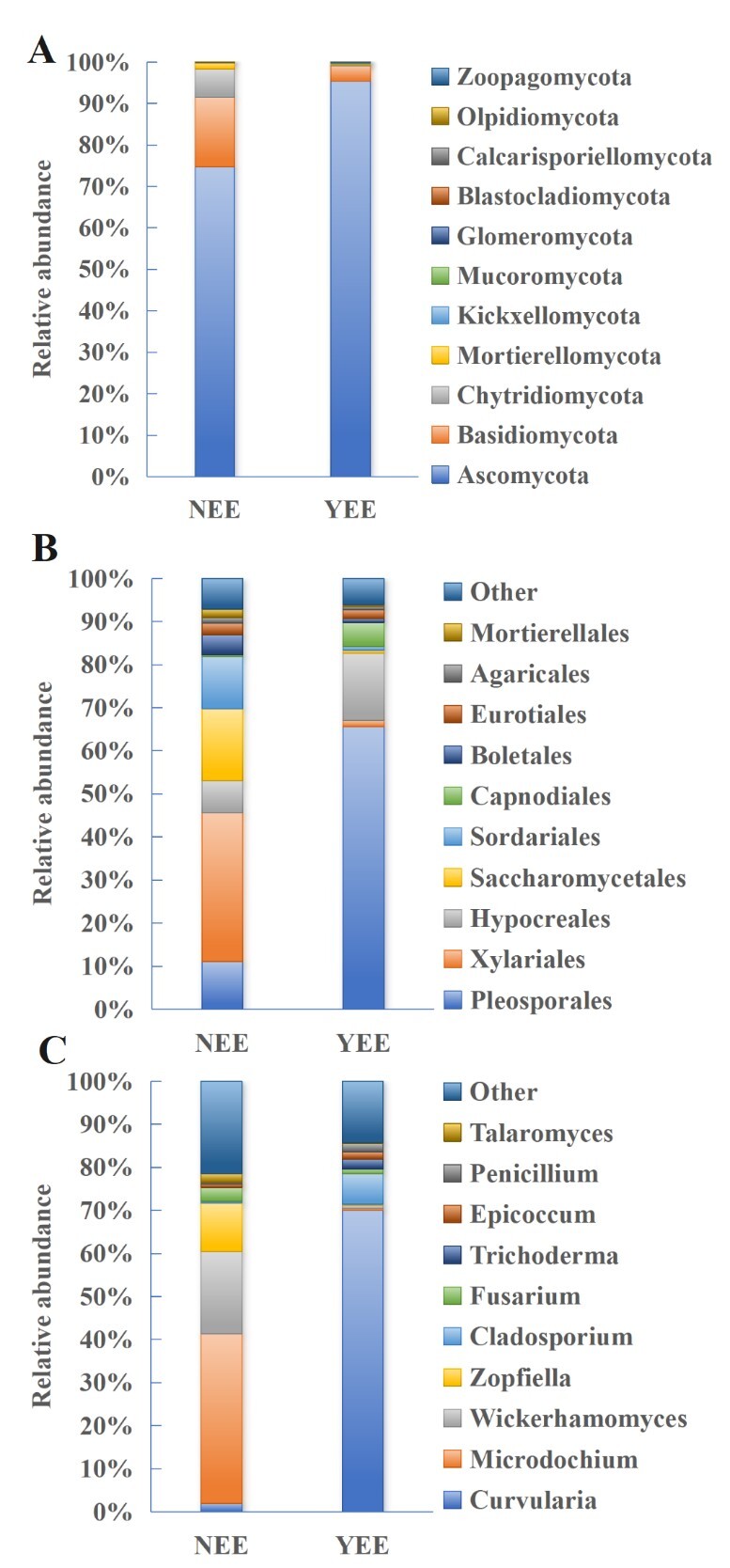
Composition of rhizosphere fungi in *C. dactylon* of NEE and YEE at the phylum (A), order (B) and genus (C) levels. The relative abundance of various fungi in NEE was the average of NEE -1, NEE -2 and NEE -3; the relative abundance of various fungi in YEE is the average of YEE -1, YEE -2 and YEE-3.

In NEE and YEE fungal communities, the most dominant phylum in *C. dactylon* rhizosphere soil is the same (Fig. 3A), both of which are Ascomycota, with relative abundances of 74.7% and 95.3%, respectively. It can be seen that the fungal community in NEE may change more to Ascomycota under the influence of eco-restoration engineering. Ascomycota is the largest fungal phylum composed of Taphrinomycotina, Saccharomycotina and Pezizomycotina with more than 8,3000 species (Shen et al. 2020). Similarly, Ascomycota was also observed to be dominant in rhizosphere soil of *Ferula sinkiangesis* (Zhang et al. 2019) and wheat (Gqozo et al. 2020, Zhang et al. 2021). Basidiomycota was the second-most fungus in NEE and YEE fungal communities, but the relative abundance was 16.8% and 3.7% with a large difference, respectively. This situation is very similar to previous findings in Rhizoma Atractylodis Macrocephalae (Song et al. 2023) and maize (Li et al. 2021), where Basidiomycota together with Ascomycota are prominent fungi in the whole fungal communities. In addition to these two fungi, Chytridiomycota (6.9%) is the dominant one in NEE, while the abundance of other fungi in YEE is close to 0.

At the class level (Suppl. material 1, Fig. S13), Dothideomycetes was found to be the most abundant in YEE (70.4%), but only accounts for 10.5% in NEE. More Sordariomycetes was found in NEE (52.3%) compared to YEE (19.0%). At the order level, fungal community composition differ greatly between NEE and YEE (Fig. 3B). Xylariales (34.4%) that contains seven families, 92 genera and 795 species (Smith et al. 2003) has an absolute dominance in NEE, but only accounts for 1.5% in YEE. Pleosporales (65.5%) and Hypocreales (15.6%) were dominant in YEE and their relative abundances in NEE were significantly reduced, which were 11.1% and 7.5%, respectively. This is similar to a previous study where Pleosporales order also largely contributes to the observed dissimilarities between conservation and conventional tillage (Wang et al. 2017). At the family level (Suppl. material 1, Fig. S14), the most abundant family is Microdochiaceae for NEE (36.6%) and Pleosporaceae for YEE (64.3%). At the genus level, fungal communities also show significant differences between NEE and YEE (Fig. 3C). *Curvularia* accounts for only 2.0% of fungal community in NEE, but it has an absolute dominance in YEE (69.9%). A previous study has reported that species could be helpful in plant growth promotion and environmental remediation (Khan et al. 2023). *Wickerhamomyces* accounted for 19.1% in NEE, but nearly 0 (0.8%) in YEE. *Zopfiella* (11.2%) accounted for a certain proportion in NEE, but nearly disappeared in YEE. *Cladosporium* accounts for 7.1% in YEE, but only 0.4% in NEE. These results suggested that eco-restoration engineering largely changed the fungal community composition at phylum, class, order, family and genus levels.

**Figure 4.**
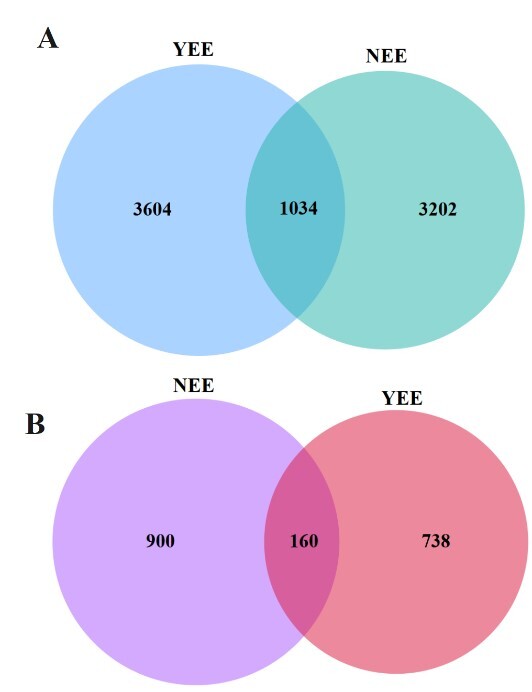
Number of common and unique ASVs in rhizosphere bacteria (A) and fungi (B) of *C. dactylon* in WLFZ of NEE and YEE.

The Chao1 indices of bacterial communities in NEE and YEE were 1718.17 ± 187.43 and 2047.40 ± 88.95, respectively (Table 1). Similarly, their observed features were 1706.00 ± 186.24 and 2030.00 ± 87.89, respectively. This indicates that the abundance of bacteria in YEE is higher than that in NEE. The Faith_pd value, Shannon value and Simpson value of YEE were higher than those of NEE. This indicates that the diversity and evenness of bacterial community are higher in YEE than in NEE. These results show that eco-restoration engineering improves the diversity of rhizosphere microorganisms in the WLFZ, so that it can better adapt to the extreme environmental conditions of WLFZ. Our previous study also observed the increase in ɑ diversity of rhizosphere microorganisms was helpful in adapting to waterlogging stress in the WLFZ of Three Gorges Reservoir (Zhou et al. 2024). The number of unique bacterial ASVs in YEE is more than that in NEE (3604 vs. 3202) and the number of bacterial ASVs shared by YEE and NEE is 1034 (Fig. 4A).

The diversity of fungal community in the rhizosphere of *C. dactylon* in the WLFZ of NEE and YEE is just opposite to the overall trend of their bacterial communities (Table 1). The Chao1 index of NEE was 434 ± 88.00, while that of YEE was 349.69 ± 162.18, with a difference of 85. This shows that eco-restoration engineering may make *C. dactylon* re-assemble the rhizosphere fungal community and reduce the richness of rhizosphere fungi. The analysis of Observed_features index, which is similar to the function of Chao1, also proves this point again. Moreover, the fungal diversity of *C. dactylon* in YEE also decreased to a certain extent, as shown by Shannon index and Simpson index, which decreased by 1.11 and 0.12, respectively compared with NEE. NEE and YEE share 160 fungal ASVs and the unique ASVs are 900 and 738, respectively (Fig. 4B). These results showed that, compared with the natural restoration area, eco-restoration engineering significantly reduced the fungal community diversity in the rhizosphere of *C. dactylon* and recruited more bacteria to adapt to the habitat of WLFZ, thus potentially supporting the growth of plants. This difference may be due to the differences in environmental adaptability between bacteria and fungi, competition for resources and interactions of *C. dactylon* with these microbes. In addition, *C. dactylon* adopted in YEE is foreign, which is different from that in NEE. This difference in host plants can alter the biotic and abiotic environments of ecosystems and, thus affects the rhizosphere fungal diversity (Gong et al. 2025).

**Key biomarkers in NEE and YEE**

**Figure 5.**
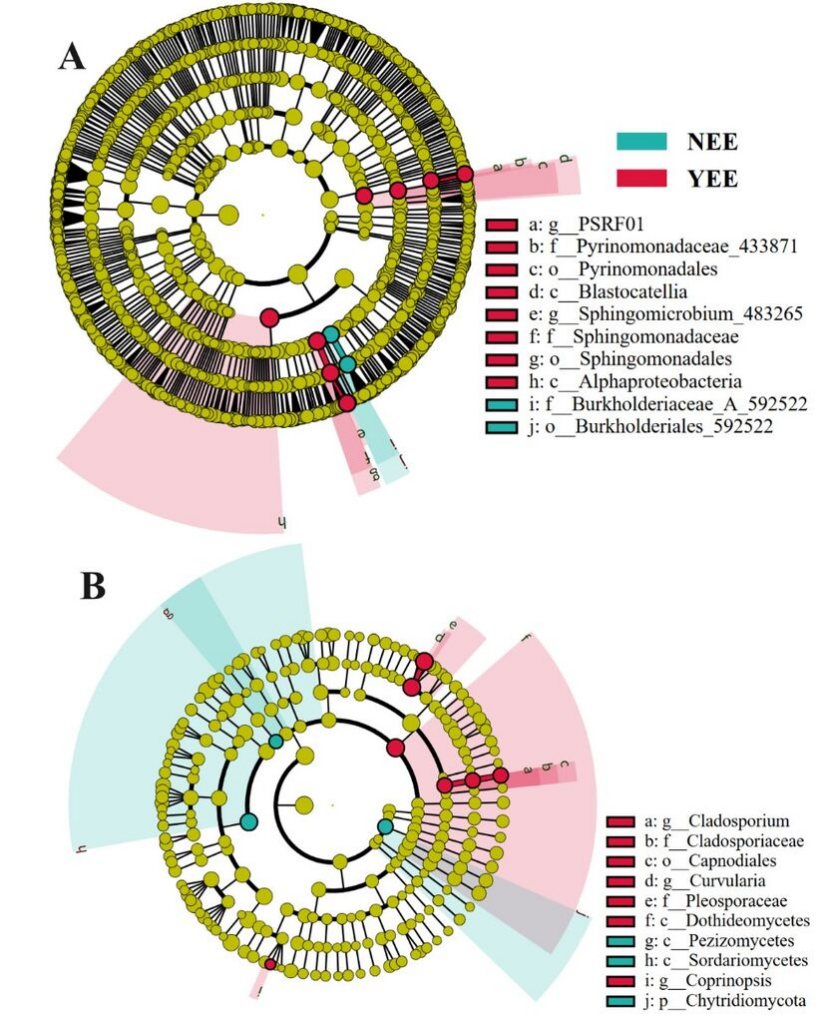
LEfSe analysis of rhizosphere bacterial (A) and fungal (B) communities of *C. dactylon* in WLFZ of NEE and YEE. PSRF01 belongs to Pyrinomonadaceae.

In this study, LEfSe analysis (LDA score ≥ 4.0) was used to investigate the biomarkers with significant differences between the microbial communities of NEE and YEE (Fig. 5). The greater the absolute value of LDA score, the more significant the contribution of this feature to the grouping difference (Khleborodova et al. 2024). In the bacterial community, the biomarkers with significant differences for YEE include two classes (Alphaproteobacteria and Blastocatellia), two orders (Pyrinomonadales and Sphingomonadales), two families (Sphingomonadaceae and Pyrinomonadaceae_433871) and two genera (*Sphingomicrobium*_483265 and PSRF01); For NEE, biomarkers with significant differences include one order (Burkholderiales_592522) and one family (Burkholderiaceae_A_592522). This indicates that YEE group shows more characteristics of high LDA scores and covers multi-level taxa from class to genus, while the NEE group has only a few characteristics significantly enriched, which is consistent with the results of α-diversity analysis. More bacterial specific biomarkers may indirectly reflect the bacterial diversity. In short, the bacterial diversity of YEE group was significantly higher than that of NEE group and involved more complex classification levels (such as class, order, family, genus); in the YEE group, Alphaproteobacteria (class) and Sphingomonadaceae (family) are significantly enriched, which may indicate that they have a core function in this group. This study also found that there were more specific biomarkers in YEE than in NEE in fungal community and the specific biomarkers in YEE included seven (1 class, Dothideomycetes; 1 order (Capnodiales), 2 families, Cladosporiaceae and Pleosporaceae; 3 genera, *Coprinopsis*, *Cladosporium* and *Curvularia*), while there are only three specific biomarkers in NEE (1 phylum, Chytridiomycota; 2 classes, Sordariomycetes and Pezizomycetes). Noteworthy, LEfSe analysis has been adopted in previous studies to determine the discriminative taxa between different soil samples from tobacco (Liu et al. 2022), alfalfa (Tang et al. 2023) and wheat (Song et al. 2020). The YEE group has more specific markers in fungal community, which may be related to its specific function or environmental stress response. The differences of phylum and class levels in NEE group may indicate more basic changes in community composition, while the differences of fungal community in the YEE group may be related to local niche adaptation. For example, the enrichment of *Curvularia* (stress tolerant fungi) and Dothideomycetes (widely distributed in complex environments) may indicate special metabolic needs or stress conditions in YEE group environment.

**Differences in microbial metabolic pathways**

**Metabolic pathways**

**Figure 6.**
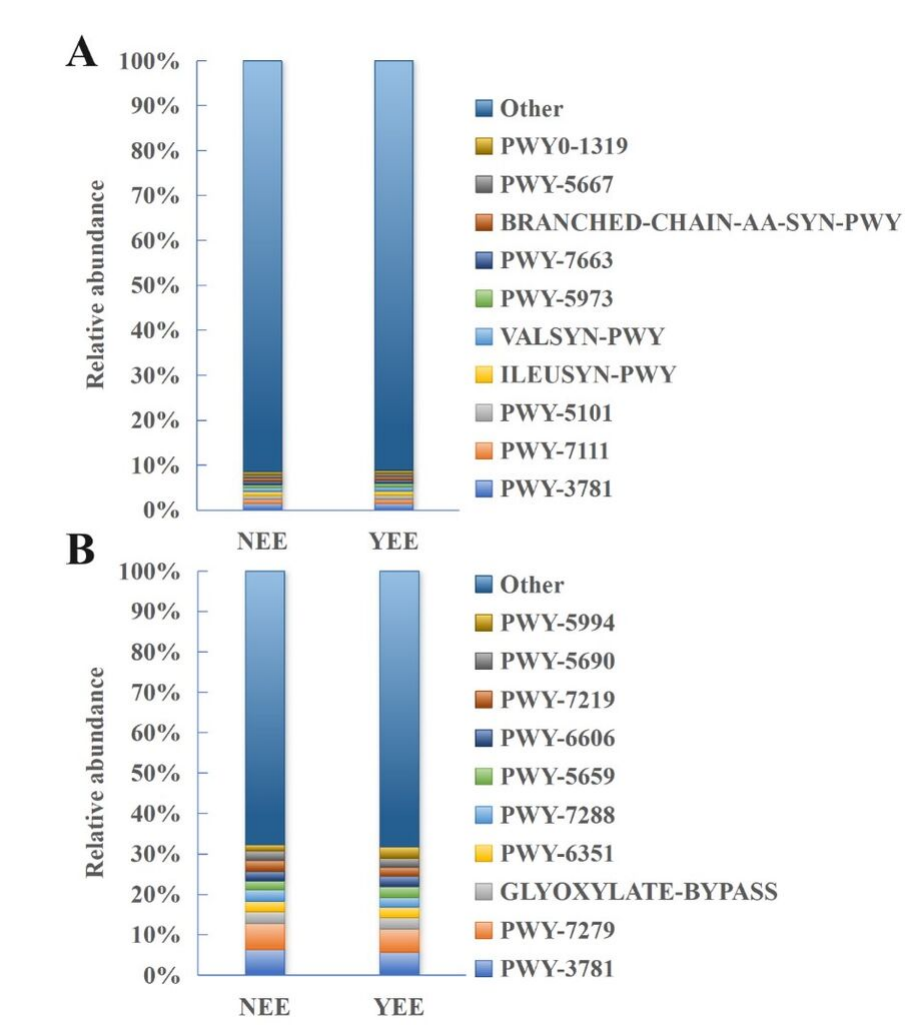
MetaCyc metabolic pathway analysis of rhizosphere soil bacteria (A) and fungi (B) of *C. dactylon* in WLFZ of NEE and YEE.

The metabolic function analysis by PICRUSt2 (Douglas et al. 2020) and MetaCyc database (Caspi et al. 2020) found that PWY-3781 [aerobic resuscitation I (cytochrome c)] had the highest relative abundance, accounting for about 1.4% in YEE and about 1.4% in NEE (Fig. 6A), which was almost the same. Interestingly, this relative abundance is almost the same as that in the rhizosphere bacterial community of *Argemone mexicana* L. at the normal growth state observed in the WLFZ of Wudongde Reservoir in the upper reaches of the Yangtze River (~1.5%) (Zhou et al. 2023). It is followed by PWY-7111 [Pyruvate fermentation to isobutanol (engineered)], PWY-5101 [L-isoleucine biosynthesis II], ILEUSYN-PWY [L-isoleucine biosynthesis I (from threonine)], VALSYN-PWY [L-valine biosynthesis], PWY-5973[cis-vaccenate biosynthesis], PWY-7663 [gondoate biosynthesis (anaerobic)], BRANCHED-CHAIN-AA-SYN-PWY [superpathway of branched chain amino acid biosynthesis], PWY-5667 [CDP-diacylglycerol biosynthesis I] and PWY0-1319 [CDP-diacylglycerol biosynthesis II]. The relative abundance of these metabolic pathways is low, only between 0.70% and 0.98%. Interestingly, this situation is very similar to that found in rhizosphere soil of Berchemia polyphylla var. leioclada where PWY–3781, PWY–7111 and PWY–5101 are top metabolic pathways with respective percentage being 1.74% - 1.77%, 0.96% - 1.07% and 1.00% - 1.05% (Tang et al. 2024). NEE and YEE are very close to each other in the composition of metabolic functions of bacterial communities. The total abundance of top 10 metabolic functions in the whole community of NEE and YEE is only 8.6% and 8.9%, respectively. Dominance of the above metabolic pathway abundance in rhizosphere bacterial community functions of *C. dactylon* in WLFZ of NEE and YEE may be related to their environmental conditions, such as nutritional restriction or metabolic pressure driving the selection of specific pathways. The “other” category accounts for a large proportion, suggesting that there may be unique features that have not been annotated, which need to be further combined with other tools for mining and analysis. Therefore, this study further performed the metabolic analyses, based on KEGG database at three levels.

**Figure 7.**
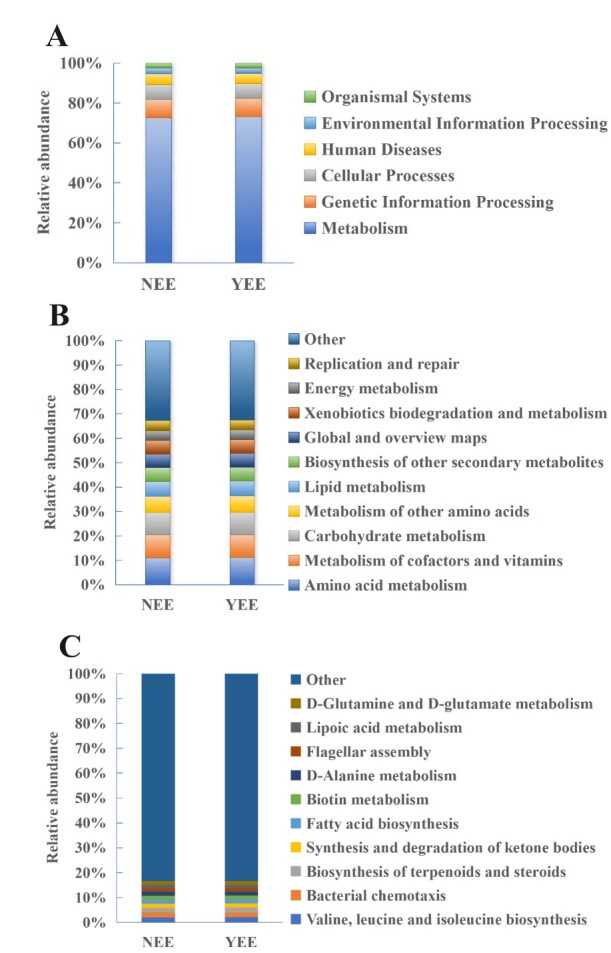
Metabolic pathway analysis of rhizosphere soil bacteria of *C. dactylon* in WLFZ of NEE and YEE at KEGG L1 (A), KEGG L2 (B) and KEGG L3 (C) levels.

At KEGG L1 level, metabolic functions are divided into metabolism, genetic information processing, cellular processes, human diseases, environmental information processing and organismal systems. Our results show that YEE is more abundant in metabolic and genetic information processing functions, but NEE is more abundant in cellular processes, human diseases, environmental information processing and biological system functions (Fig. 7A). These results showed that YEE group showed stronger metabolic activity and lower environmental response ability, which may reflect the regulatory effect of environmental pressure on microbial community function. The KEGG-L1-level functional profile analysis also showed that metabolism and genetic information processing are key functions in rhizosphere microbes of five tea cultivars (Wu et al. 2025), as observed in YEE group.

At KEGG L2 level, metabolic functions include xenobiotics biodegradation and metabolism, carbohydrate metabolism, amino acid metabolism, metabolism of cofactors and vitamins, global and overview maps. Amino acid metabolism accounted for the highest proportion, followed by co-factors and vitamin metabolism in NEE and YEE (Fig. 7B). This is somewhat different from that was observed for five tea cultivars where carbohydrate metabolism accounts for the highest proportion (Wu et al. 2025). The YEE group was more active in energy metabolism (such as carbohydrate metabolism) and basic metabolism (such as amino acid metabolism), while the NEE group was more inclined to environmental adaptation related functions (such as Xenobiotics biodegradation and metabolism).

At KEGG L3 level (Fig. 7C), metabolic pathways are involved in D-Glutamine and D-glutamate metabolism, lipoic acid metabolism, flagellar assembly, D-Alanine metabolism, biotin metabolism, fatty acid biosynthesis, synthesis and degradation of ketone bodies, biosynthesis of terpenoids and steroids, bacterial chemotaxis and valine, leucine and isoleucine biosynthesis. The most abundant pathways in YEE were valine, leucine and isoleucine biosynthesis (2.08%), biosynthesis of terpenoids and steroids (1.96%) and bacterial chemotaxis (1.85%), while the most abundant pathways in NEE were bacterial chemotaxis (2.07%), valine, leucine and isoleucine biosynthesis (2.01%) and synthesis and degradation of ketone bodies (1.79%). The enrichment of amino acid metabolism, terpenoids and steroids biosynthesis pathway in YEE group may reflect the enhancement of microbial environmental adaptability, while the bacterial chemotaxis and ketone synthesis and degradation in NEE group may be related to their specific physiological needs. Previous studies also investigated the KEGG-L3-level functional profiles of rhizosphere bacteria in different plants and found that the KEGG-L3-level functional profiles varied largely between different plants (e.g. *Panax notoginseng* (Kui et al. 2021), tea (Wu et al. 2025) and *Bletilla striata* (Thunb.) Reichb. f. (Li et al. 2024)).

In addition, this study also analysed the metabolic functions of NEE and YEE fungal communities (Fig. 6B) and found that the most dominant metabolic pathways of both are PWY-3781 and PWY-7279 [aerial resuscitation II (cytochrome c) (yeast)], which are basically the same as the rhizosphere fungal communities of *Argemone mexicana* L. in the WLFZ of Wudongde Reservoir (Zhou et al. 2023). These results indicate that, under extreme stress in the WLFZ, plant rhizosphere fungi tend to enrich metabolic pathways related to aerobic respiration to adapt to their survival. The stable proportion of the core energy metabolism pathway (PWY-3781) also reflects the importance of its basic physiological functions. Compared with NEE group, the microbial community in YEE group was enriched with more GLYOXYLATE-BYPASS, suggesting that metabolic activity may be used to adapt to specific carbon source constraints. The relative abundance of PWY-5994 [palmitate biosynthesis I (type I fatty acid synthase)] was significantly different between NEE and YEE, which were 1.6% and 2.8%, respectively. The higher abundance of PWY-5994 pathway in YEE group may show its enhanced ability to biosynthesis of palmitic acid. In addition to PWY-5994, the relative abundances of the remaining eight top metabolic pathways were also very close. Interestingly, the total abundance of top 10 metabolic pathway in fungal community was significantly higher than that of bacterial community, accounting for 32.3% and 31.7% of NEE and YEE, respectively.

**Enzyme functional characteristics**

**Figure 8.**
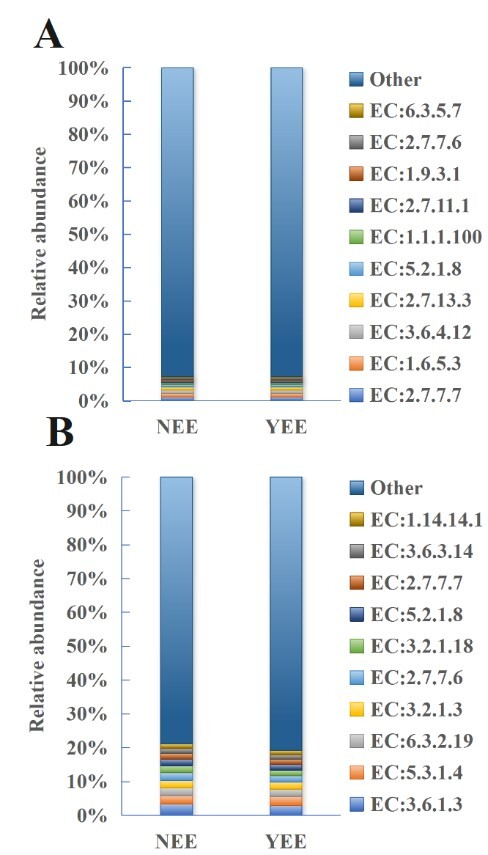
Enzyme functional analysis of rhizosphere soil bacteria (A) and fungi (B) of *C. dactylon* in WLFZ of NEE and YEE. EC:2.7.7.7, DNA-directed DNA polymerase; EC:1.6.5.3, NADH:ubiquinone reductase (H(+)-translocating); EC:3.6.4.12, DNA helicase; EC:2.7.13.3, histidine kinase; EC:5.2.1.8, peptidylprolyl isomerase; EC:1.1.1.100, 3-oxoacyl-[acyl-carrier-protein] reductase; EC:2.7.11.1, non-specific serine/threonine protein kinase; EC:1.9.3.1, cytochrome-c oxidase; EC:3.6.1.3, phosphoribosyl-ATP diphosphatase; EC:6.3.5.7, glutaminyl-tRNA synthase (glutamine-hydrolyzing); EC:5.3.1.4, L-arabinose isomerase; EC:3.2.1.3, Glucan 1,4-alpha-glucosidase; EC:2.7.7.6, DNA-directed RNA polymerase; EC:3.2.1.18, Exo-alpha-sialidase; EC:3.6.3.14, H(+)-transporting two-sector ATPase; EC:6.3.2.19, ubiquitin-protein ligase; EC:5.2.1.8, peptidylprolyl isomerase; EC:1.14.14.1, unspecific monooxygenase.

The functional characteristics of *C. dactylon* rhizosphere bacterial enzymes in NEE and YEE groups showed that the most dominant enzyme was EC:2.7.7.7 (DNA-directed DNA polymerase), but its advantages are not particularly prominent and its relative abundance was only about 1.1%, followed by EC:1.6.5.3 (NADH:ubiquinone reductase (H(+)-translocating)) and EC:3.6.4.12 (DNA helicase) with their relative abundance exceeding 1% (Fig. 8A). Interestingly, these three enzymes were also found in the most enriched functions in the rhizosphere microbes of *Artemisia frigida*, *Acorus tatarionwii* Schott. and *Salix oritrepha* Schneid (Tang et al. 2024a). The YEE group showed stronger nucleic acid synthesis related function (e.g. EC:2.7.7.7) and energy metabolism activity (e.g. EC:1.6.5.3), while EC:2.7.13.3 (histidine kinase), EC:5.2.1.8 (peptidylprolyl isomerase), EC:2.7.11.1 (Non-specific serine/threonine protein kinase) and EC:1.9.3.1 (cytochrome-c oxidase) are more prominent in the NEE group, suggesting that eco-restoration engineering may play a directional role in regulating the functional spectrum of microbial communities.

Fig. 8B displays the relative abundance distribution of enzyme functional characteristics (EC numbers) within the rhizosphere fungal community of *C. dactylon*, which ranks highest in total abundance, across different groups (NEE and YEE). The study reveals that EC:3.6.1.3 (phosphoribosyl-ATP diphosphatase) is the enzyme with the highest relative abundance in both NEE and YEE, accounting for 3.48% and 2.99%, respectively. For EC:5.3.1.4 (L-arabinose isomerase), EC:6.3.2.19, EC:3.2.1.3 (Glucan 1,4-alpha-glucosidase), EC:2.7.7.6 (DNA-directed RNA polymerase), EC:3.2.1.18 (Exo-alpha-sialidase), EC:5.2.1.8, EC:2.7.7.7 and EC:3.6.3.14 (H(+)-transporting two-sector ATPase), the enzyme abundance in NEE is higher than that in YEE. EC:1.14.14.1 stands as the sole exception, with a relative abundance of 1.41% in YEE, surpassing the 1.32% in NEE. The "Other" category comprises a higher proportion in the YEE group (80.71% vs. 78.88%), potentially indicating a more intricate functional diversity.

**Analysis of ecological function**

**Figure 9.**
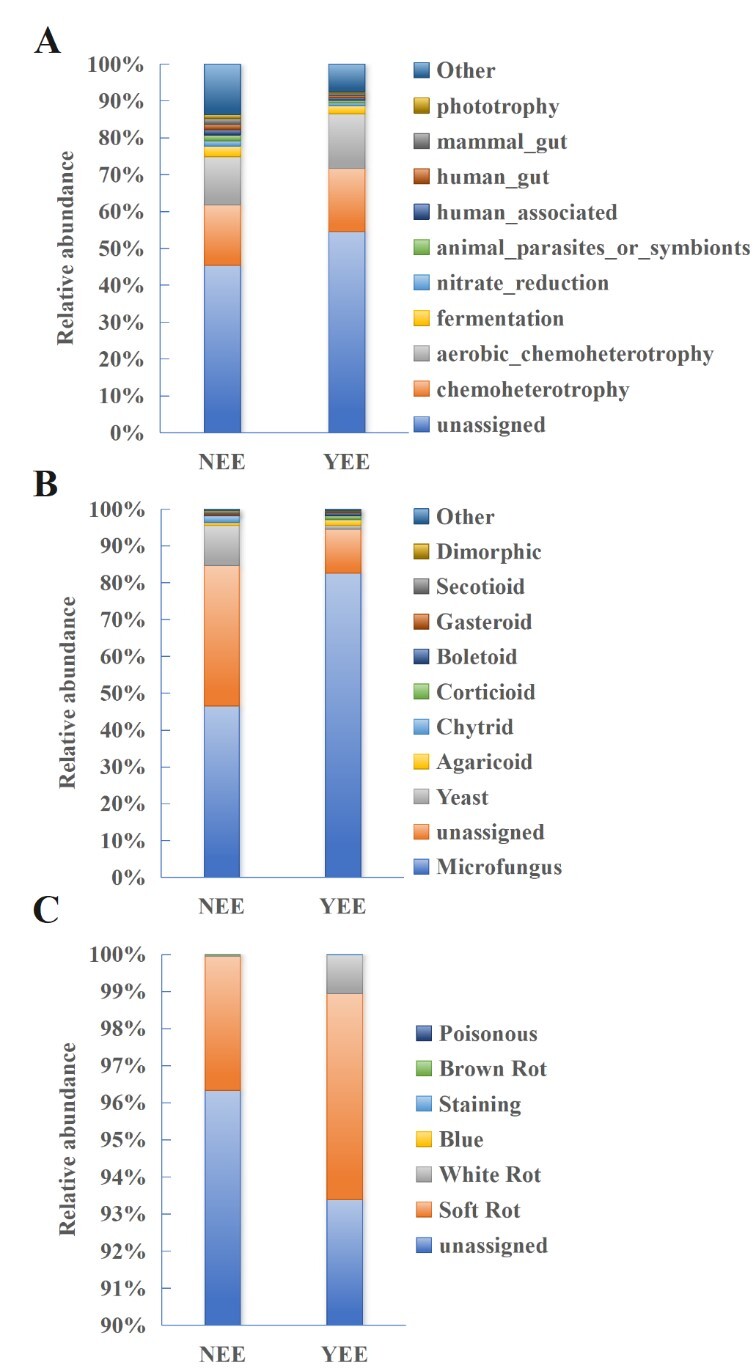
Ecological function analysis of rhizosphere soil bacteria (A) and fungi (B, growth; C, trait) of *C. dactylon* in WLFZ of NEE and YEE.

In this study, FAPROTAX (Functional Annotation of Prokaryotic Taxa) (Sansupa et al. 2021) was used to analyse the ecological function of rhizosphere bacteria in YEE and NEE groups (Fig. 9A). Chemoheterotrophy accounted for a higher proportion in YEE group (~ 17.23% vs. NEE ~ 16.41%). Ke et al. (2023) also found chemoheterotrophy related to carbon cycle had the highest relative abundance in the rhizosphere of *Commelina communis*. The second is aerobic_chemoheterotrophy, which accounts for 14.73% and 13.01% in YEE and NEE, respectively. These results indicated that the energy metabolism of rhizosphere bacteria in *C. dactylon* was more active in the eco-restoration engineering area. Fermentation, nitrate_reduction, animal_parasites_or_symbionts, human_associated and phototrophy have a higher percentage in NEE group which may be related to organic matter decomposition, element cycle, environment or host interaction. Unclassified functions accounted for the highest proportion in the two groups (NEE ~ 45.39%, YEE ~ 54.51%), which may reflect a large number of uncommented or unknown functional features.

Using FUNGuild (Nguyen et al. 2016), microfungus was the absolute dominant one in both NEE and YEE groups (Fig. 9B), but the proportion of YEE group (about 82.62%) was significantly higher than that of NEE group (about 46.54%). Yeast accounted for about 10.81% in NEE group, but only 1.03% in YEE group. Agaricoid accounted for about 1.46% in YEE group and only 0.88% in NEE group. The proportion of boletoid and corticoid in YEE group was higher than that in NEE group. The YEE group was significantly enriched in microfungus where their proportion was significantly increased and other groups were strongly inhibited. The fungal community of NEE group was diversified and the microfungus, yeast, agaricoid and other groups co-existed and the community structure was more complex. Amongst the identified fungal functional characteristics, the microbial functional characteristics of YEE group are dominated by soft-rot fungi and white-rot fungi, which may be related to specific metabolic activities (such as cellulose and lignin degradation) (Fukasawa 2021). The NEE group was dominated by soft-rot fungi (Fig. 9C). Soft-rot and white-rot fungi are two types of the best-known wood-decay fungi and they can work together to decompose organic matter in the soil, for example, lignin, cellulose and hemicellulose (Sista Kameshwar and Qin 2017).

**Network analysis**

**Figure 10.**
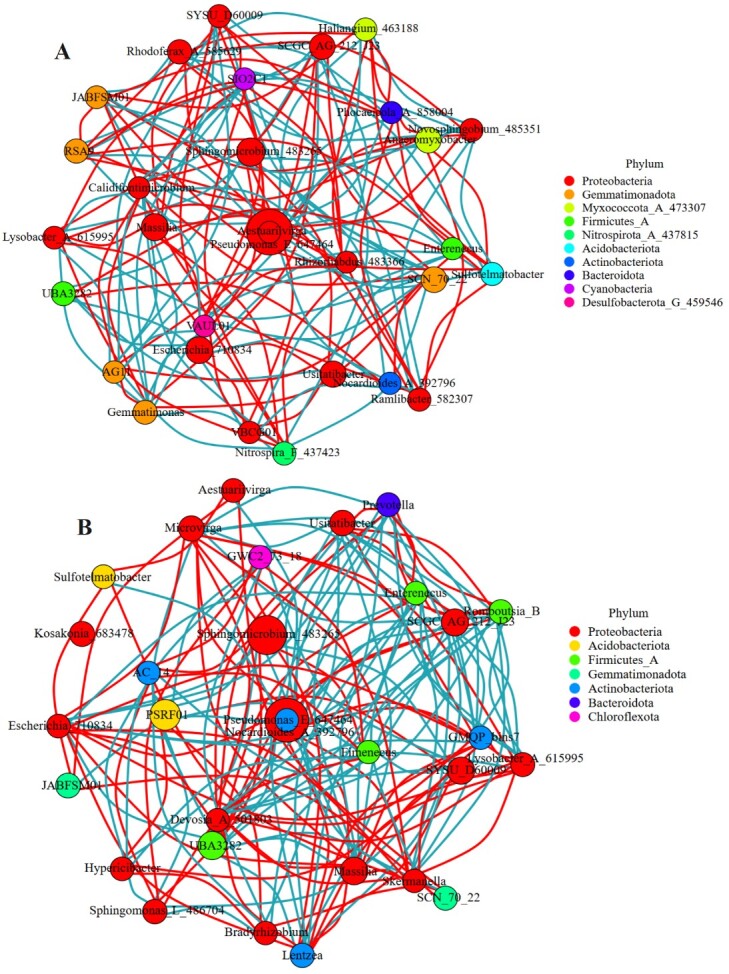
Network analysis of rhizosphere soil bacteria of *C. dactylon* in WLFZ of NEE (A) and YEE (B).

**Figure 11.**
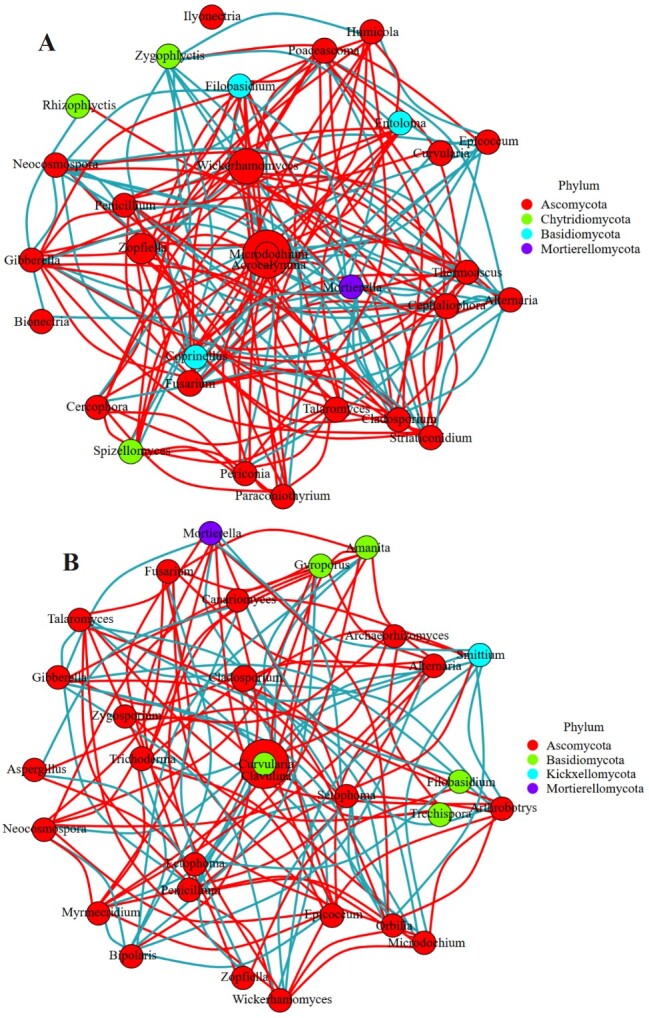
Network analysis of rhizosphere soil fungi of *C. dactylon* in WLFZ of NEE (A) and YEE (B).

Network analysis is often used to explore microbial interactions (Sui et al. 2023, Niu et al. 2024). By calculating the correlation between species, the network analysis diagram of species was established at the genus level. The results showed that 142 positive or negative interactions existed in the bacterial community of NEE, of which 75 were negative and 67 were positive (Fig. 10A); in the YEE group, the number of interactions increased to 151, including 78 negative and 73 positive interactions (Fig. 10B). For fungal communities, the YEE group found 116 pairs of positive or negative interactions amongst fungal genera, including 50 negative associations and 66 positive associations (Fig. 11B); the number of interactions in the NEE group was significantly higher than that in the YEE group, reaching 163 and the positive and negative interactions were 99 and 64, respectively (Fig. 11A). These results showed that eco-restoration engineering increased the interactions between bacteria in the rhizosphere of *C. dactylon*, but inhibited the interactions between some fungi, which may be linked to the changes of microbial ecological functionality caused by YEE. Microbial interactions are believed to play a more important role in ecological functionality than the diversity and abundance of microorganisms (Zhao et al. 2022).

The main nodes in the rhizosphere bacterial network in the natural recovery area are Proteobacteria, Gemmatimonadota, Myxococcota_A_473307, Firmicutes_A, Nitrospirota_A_437815, Acidobacteriota, Actinobacteriota, Bacteroidota, Cyanobacteria and Desulfobacterota_G_459546, while the interactions in fungal communities are mainly concentrated amongst Ascomycota, Chytridiomycota, Basidiomycota and Mortierellomycota. The main nodes in the rhizosphere bacterial network in the eco-restoration engineering area are involved in Proteobacteria, Acidobacteriota, Firmicutes_A, Gemmatimonadota, Actinobacteriota, Bacteroidota and Chloroflexota and the interactions of rhizosphere fungal communities mainly appear amongst Ascomycota, Basidiomycota, Kickxellomycota and Mortierellomycota. These results showed all these bacterial or fungal interaction networks in YEE or NEE groups were complex and eco-restoration engineering changed their interaction networks, but always maintained the co-existing relationship between bacteria and fungi. It has been reported that the co-existence of these two types of microbes are generally more stable than the single existence of them (Gong et al. 2025).

## Conclusions

In this study, the rhizosphere microorganisms of *C. dactylon*, a typical dominant plant in NEE and YEE, were investigated through experimental design and the effects of eco-restoration engineering on the structural diversity, composition and ecological function of rhizosphere bacterial and fungal communities of typical dominant plant were clarified through comparative analysis. Our study showed that the dominant bacterial and fungal phyla in the rhizosphere microbial community of *C. dactylon* in NEE and YEE was Proteobacteria and Ascomycota, respectively. The α-diversity of rhizosphere bacteria in YEE was significantly higher than that in NEE, but this situation is opposite for rhizosphere fungi. Eco-restoration engineering did not change the dominant position of key metabolic pathways in the microbial communities in NEE and YEE. Many microbial groups (such as Alphaproteobacteria and Sphingomonadaceae) in YEE were identified as significantly enriched biomarkers. In contrast, the key biomarkers in the rhizosphere in NEE are relatively few and Burkholderiaceae and Burkholderiales may be the key characteristic microbe. The present study provides new insight into the in-depth understanding the role of eco-restoration engineering in the re-assembly of rhizosphere microorganisms and supporting vegetation restoration in the WLFZ of large river-type reservoirs. Future research can consider to explore the interaction mechanisms between rhizosphere microbes and plants in the WLFZ and integrate multi-omics (e.g. metagenomics and metabolomics) to accelerate the development of functional microbial agents for guiding ecological restoration of WLFZ.

## Supplementary Material

0D0D6E7E-12F6-5427-B8EA-57B5BA20CE3B 10.3897/BDJ.13.e159524.suppl1 Supplementary material 1 Supplementary file Data type Composition and their variation of rhizosphere microbes in *C. dactylon*. File: oo_1447907.docx https://binary.pensoft.net/file/1447907 Lanfang Zhou, Shengjun Wu, Maohua Ma

## Figures and Tables

**Table 1.** Alpha diversity indices of rhizosphere microbial community of *C. dactylon* in WLFZ of NEE and YEE

Sample	Microbe	Chao1 ± sd	Faith_pd ± sd	Observed_features ± sd	Shannon ± sd	Simpson ± sd
NEE	Bacteria	1718.17 ± 187.43	100.05 ± 12.76	1706.00 ± 186.24	9.44 ± 0.11	0.994 ± 0.003
	Fungi	434 ± 88.00	92.41 ± 21.48	434.00 ± 88.00	4.76 ± 1.24	0.809 ± 0.167
YEE	Bacteria	2047.40 ± 88.95	117.26 ± 7.44	2030.00 ± 87.89	9.85 ± 0.11	0.996 ± 0.002
	Fungi	349.69 ± 162.18	68.67 ± 25.57	349.67 ± 162.14	3.65 ± 1.46	0.691 ± 0.197

## References

[B13386156] Berg Jeanette, Brandt Kristian K., Al-Soud Waleed A., Holm Peter E., Hansen Lars H., Sørensen Søren J., Nybroe Ole (2012). Selection for Cu-tolerant bacterial communities with altered composition, but unaltered richness, via long-term Cu exposure. Applied and Environmental Microbiology.

[B13394291] Bolyen Evan, Rideout Jai Ram, Dillon Matthew R, Bokulich Nicholas A, Abnet Christian C, Al-Ghalith Gabriel A, Alexander Harriet, Alm Eric J, Arumugam Manimozhiyan, Asnicar Francesco, Bai Yang, Bisanz Jordan E, Bittinger Kyle, Brejnrod Asker, Brislawn Colin J, Brown C Titus, Callahan Benjamin J, Caraballo-Rodríguez Andrés Mauricio, Chase John, Cope Emily K, Da Silva Ricardo, Diener Christian, Dorrestein Pieter C, Douglas Gavin M, Durall Daniel M, Duvallet Claire, Edwardson Christian F, Ernst Madeleine, Estaki Mehrbod, Fouquier Jennifer, Gauglitz Julia M, Gibbons Sean M, Gibson Deanna L, Gonzalez Antonio, Gorlick Kestrel, Guo Jiarong, Hillmann Benjamin, Holmes Susan, Holste Hannes, Huttenhower Curtis, Huttley Gavin A, Janssen Stefan, Jarmusch Alan K, Jiang Lingjing, Kaehler Benjamin D, Kang Kyo Bin, Keefe Christopher R, Keim Paul, Kelley Scott T, Knights Dan, Koester Irina, Kosciolek Tomasz, Kreps Jorden, Langille Morgan G I, Lee Joslynn, Ley Ruth, Liu Yong-Xin, Loftfield Erikka, Lozupone Catherine, Maher Massoud, Marotz Clarisse, Martin Bryan D, McDonald Daniel, McIver Lauren J, Melnik Alexey V, Metcalf Jessica L, Morgan Sydney C, Morton Jamie T, Naimey Ahmad Turan, Navas-Molina Jose A, Nothias Louis Felix, Orchanian Stephanie B, Pearson Talima, Peoples Samuel L, Petras Daniel, Preuss Mary Lai, Pruesse Elmar, Rasmussen Lasse Buur, Rivers Adam, Robeson Michael S, Rosenthal Patrick, Segata Nicola, Shaffer Michael, Shiffer Arron, Sinha Rashmi, Song Se Jin, Spear John R, Swafford Austin D, Thompson Luke R, Torres Pedro J, Trinh Pauline, Tripathi Anupriya, Turnbaugh Peter J, Ul-Hasan Sabah, van der Hooft Justin J J, Vargas Fernando, Vázquez-Baeza Yoshiki, Vogtmann Emily, von Hippel Max, Walters William, Wan Yunhu, Wang Mingxun, Warren Jonathan, Weber Kyle C, Williamson Charles H D, Willis Amy D, Xu Zhenjiang Zech, Zaneveld Jesse R, Zhang Yilong, Zhu Qiyun, Knight Rob, Caporaso J Gregory (2019). Reproducible, interactive, scalable and extensible microbiome data science using QIIME 2.. Nature biotechnology.

[B12964912] Caspi Ron, Billington Richard, Keseler Ingrid M, Kothari Anamika, Krummenacker Markus, Midford Peter E, Ong Wai Kit, Paley Suzanne, Subhraveti Pallavi, Karp Peter D (2020). The MetaCyc database of metabolic pathways and enzymes - a 2019 update.. Nucleic acids research.

[B13395396] Chang Fang, He Shishi, Dang Chenyuan (2022). Assisted selection of biomarkers by Linear Discriminant Analysis Effect Size (LEfSe) in microbiome data. Journal of Visualized Experiments.

[B12964927] Chen Yan, Tian Wei, Shao Yang, Li Ying-Jun, Lin Li-An, Zhang Ying-Jun, Han Hui, Chen Zhao-Jin (2020). Miscanthus cultivation shapes rhizosphere microbial community structure and function as assessed by Illumina MiSeq sequencing combined with PICRUSt and FUNGUIld analyses.. Archives of Microbiology.

[B12966046] Dong Zhou-Yan, Rao Manik Prabhu Narsing, Liao Tian-Jiang, Li Li, Liu Yong-Hong, Xiao Min, Mohamad Osama Abdalla Abdelshafy, Tian Yue-Ying, Li Wen-Jun (2021). Diversity and function of rhizosphere microorganisms between wild and cultivated medicinal plant *Glycyrrhiza
uralensis* Fisch under different soil conditions.. Archives of Microbiology.

[B12964899] Douglas Gavin M., Maffei Vincent J., Zaneveld Jesse R., Yurgel Svetlana N., Brown James R., Taylor Christopher M., Huttenhower Curtis, Langille Morgan G. I. (2020). PICRUSt2 for prediction of metagenome functions. Nature Biotechnology.

[B13596044] Fukasawa Yu (2021). Ecological impacts of fungal wood decay types: A review of current knowledge and future research directions. Ecological Research.

[B12964855] Gao Wei, Chen Shuyi, Yu Xin, Chen Sumin, Wan Caijing, Wang Ying, Wu Peng, Li Qiang (2025). Three local plants adapt to ecological restoration of abandoned lead-zinc mines through assembly of rhizosphere bacterial communities. Frontiers in Microbiology.

[B13381075] Gao Yunyun, Zhang Guoxing, Jiang Shunyao, Liu Yong-Xin (2024). Wekemo Bioincloud: A user-friendly platform for meta-omics data analyses.. iMeta.

[B13592872] Gong Mengxin, Wang Jilian, Li Mingyuan (2025). Plant species shaping rhizosphere fungal community structure in the subalpine forest steppe belt. Rhizosphere.

[B12966165] Gqozo Magalane Pheladi, Bill Malick, Siyoum Nazareth, Labuschagne Nico, Korsten Lise (2020). Fungal diversity and community composition of wheat rhizosphere and non-rhizosphere soils from three different agricultural production regions of South Africa. Applied Soil Ecology.

[B13587651] Griffiths Robert I., Whiteley Andrew S., O'Donnell Anthony G., Bailey Mark J. (2000). Rapid method for coextraction of DNA and RNA from natural environments for analysis of ribosomal DNA- and rRNA-based microbial community composition. Applied and Environmental Microbiology.

[B12964764] Jiang Weiwei, Li Wentao, Zhou Jianguo, Wang Pengcheng, Xiao Henglin (2022). Drone-based investigation of natural restoration of vegetation in the water level fluctuation zone of cascade reservoirs in Jinsha River. Scientific Reports.

[B12964868] Jin Ke, Lu Yang, Zhang Qianzhu, Wan Dan, Wu Yihang, Hu Yue (2025). Ecological restoration in water-level-fluctuation zone of reservoir in the lower reaches of Jinsha River Basin. Ecology and Environmental Monitoring of Three Gorges.

[B12966258] Ke Tan, Wang Huan, Li Shaofeng, Zhang Yurui, Wang Panpan, Chen Chaoqi, Lu Lu, Chen Lanzhou (2023). Belowground facilitation of plant mixtures on rhizosphere soil of *Commelina
communis* grown on extremely Cu- and Cd-contaminated mine: From soil quality to bacterial community. Applied Soil Ecology.

[B13394014] Khan N. A, Asaf S., Ahmad W., Jan R., Bilal S., Khan I., Khan A. L., Kim K. - M., Al-Harrasi A. (2023). Diversity, lifestyle, genomics, and their functional role of *Cochliobolus*, *Bipolaris*, and *Curvularia* species in environmental remediation and plant growth promotion under biotic and abiotic tressors. Journal of Fungi.

[B13592986] Khleborodova Asya, Gamboa-Tuz Samuel D, Ramos Marcel, Segata Nicola, Waldron Levi, Oh Sehyun (2024). lefser: implementation of metagenomic biomarker discovery tool, LEfSe, in R. Bioinformatics.

[B13595209] Kui Ling, Chen Baozheng, Chen Jian, Sharifi Rouhallah, Dong Yang, Zhang Zhanjiang, Miao Jianhua (2021). A comparative analysis on the structure and function of the *Panax
notoginseng* rhizosphere microbiome. Frontiers in Microbiology.

[B12964813] Lan Qing, Liu Guanzhi, Song Haifeng, Liu Guohou, Xu Xiao (2025). Plant sex alters rhizosphere microorganisms assembly of *Salix
gordejevii* across diverse sandy habitats. Plant and Soil.

[B13393707] Lei Shaonan, Xu Xiaohong, Cheng Zhiqiang, Xiong Juan, Ma Rongqin, Zhang Lanlan, Yang Xiaorong, Zhu Yunxi, Zhang Binghuo, Tian Baoyu (2018). Analysis of the community composition and bacterial diversity of the rhizosphere microbiome across different plant taxa. MicrobiologyOpen.

[B12964844] Li Jichao, Xu Zongliang, Yang Tianmei, Zhang Jinyu, Zuo Yingmei, Cheng Lei (2025). Rhizosphere ecological restoration: interactions between nutrient mobilization, core microbial assembly, and phenylalanine metabolism circulation. Biochar.

[B13595221] Li Shiqing, Li Xiaomei, Ye Yueyu, Chen Man, Chen Haimin, Yang Dongfeng, Li Meiya, Jiang Fusheng, Zhang Xiaobo, Zhang Chunchun (2024). The rhizosphere microbiome and its influence on the accumulation of metabolites in *Bletilla
striata* (Thunb.) Reichb. f. BMC Plant Biology.

[B12964774] Liu Jiahao, Qian Yan, Yang Wanqing, Yang Meihua, Zhang Yue, Duan Baozhong, Yang Yongcheng, Tao Aien, Xia Conglong (2024). Elucidating the interaction of rhizosphere microorganisms and environmental factors influencing the quality of *Polygonatum
kingianum* Coll. et Hemsl.. Scientific Reports.

[B12966207] Liu Xiaojiao, Liu Liehua, Gong Jie, Zhang Lixin, Jiang Qipeng, Huang Kuo, Ding Wei (2022). Soil conditions on bacterial wilt disease affect bacterial and fungal assemblage in the rhizosphere.. AMB Express.

[B12964834] Li Wenjing, Wang Hengfang, Lv Guanghui, Wang Jinlong, Li Jianhao (2024). Regulation of drought stress on nutrient cycle and metabolism of rhizosphere microorganisms in desert riparian forest.. Science of the Total environment.

[B12966196] Li Y. N., Wang T. Y., Wang C. Y., Li M. S., Wang Y., Liu S. X. (2021). Responses of soil rhizosphere fungi to N application levels in different types of soil. Applied Ecology and Environmental Research.

[B13386116] Li Zhigang, Zu Chao, Wang Can, Yang Jianfeng, Yu Huan, Wu Huasong (2016). Different responses of rhizosphere and non-rhizosphere soil microbial communities to consecutive *Piper
nigrum* L. monoculture. Scientific Reports.

[B13589368] Malviya Mukesh Kumar, Li Chang-Ning, Lakshmanan Prakash, Solanki Manoj Kumar, Wang Zhen, Solanki Anjali Chandrol, Nong Qian, Verma Krishan K., Singh Rajesh Kumar, Singh Pratiksha, Sharma Anjney, Guo Dao-Jun, Dessoky Eldessoky S., Song Xiu-Peng, Li Yang-Rui (2022). High-throughput sequencing-based analysis of rhizosphere and diazotrophic bacterial diversity among wild progenitor and closely related species of sugarcane (*Saccharum* spp. inter-specific hybrids). Frontiers in Plant Science.

[B13386146] Michelsen Charlotte Frydenlund, Pedas Pai, Glaring Mikkel Andreas, Schjoerring Jan Kofod, Stougaard Peter (2013). Bacterial diversity in Greenlandic soils as affected by potato cropping and inorganic versus organic fertilization. Polar Biology.

[B12966092] Nguyen Nhu H., Song Zewei, Bates Scott T., Branco Sara, Tedersoo Leho, Menke Jon, Schilling Jonathan S., Kennedy Peter G. (2016). FUNGuild: An open annotation tool for parsing fungal community datasets by ecological guild. Fungal Ecology.

[B13393269] Niu Hongjin, Yuan Min, Chen Xiaobo, Zhao Jingwei, Cui Yushuang, Song Yao, Zhou Sihao, Song Alin, Huang Yali (2024). Deciphering the differences of bacterial communities between high- and low-productive wheat fields using high-throughput sequencing.. Frontiers in microbiology.

[B12966081] Sansupa Chakriya, Wahdan Sara Fareed Mohamed, Hossen Shakhawat, Disayathanoowat Terd, Wubet Tesfaye, Purahong Witoon (2021). Can we use functional annotation of prokaryotic taxa (FAPROTAX) to assign the ecological functions of soil bacteria?. Applied Sciences.

[B12966106] Sharma Vaibhav, Vashishtha Amit, Jos Arsha Liz M., Khosla Akshita, Basu Nirmegh, Yadav Rishabh, Bhatt Amit, Gulani Akshanshi, Singh Pushpa, Lakhera Sanidhya, Verma Mansi (2022). Phylogenomics of the phylum Proteobacteria: Resolving the complex relationships. Current Microbiology.

[B12966140] Shen Xing-Xing, Steenwyk Jacob L, LaBella Abigail L, Opulente Dana A, Zhou Xiaofan, Kominek Jacek, Li Yuanning, Groenewald Marizeth, Hittinger Chris T, Rokas Antonis (2020). Genome-scale phylogeny and contrasting modes of genome evolution in the fungal phylum Ascomycota.. Science Advances.

[B13596085] Sista Kameshwar Ayyappa Kumar, Qin Wensheng (2017). Comparative study of genome-wide plant biomass-degrading CAZymes in white rot, brown rot and soft rot fungi. Mycology.

[B13393887] Smith G. J., Liew E. C.Y., Hyde K. D. (2003). The Xylariales: A monophyletic order containing 7 families. Fungal Diversity.

[B12966229] Song Li, Pan Zhenzhi, Dai Yi, Chen Lin, Zhang Li, Liao Qilin, Yu Xiezhi, Guo Hongyan, Zhou Guisheng (2020). Characterization and comparison of the bacterial communities of rhizosphere and bulk soils from cadmium-polluted wheat fields.. PeerJ.

[B12966185] Song Pingping, Liu Junling, Huang Peng, Han Zhili, Wang Dianlei, Sun Nianxia (2023). Diversity and structural analysis of rhizosphere soil microbial communities in wild and cultivated Rhizoma Atractylodis Macrocephalae and their effects on the accumulation of active components.. PeerJ.

[B12966131] Spain Anne M, Krumholz Lee R, Elshahed Mostafa S (2009). Abundance, composition, diversity and novelty of soil Proteobacteria.. The ISME journal.

[B13393283] Sui Jinlei, He Xiaowen, Yi Guohui, Zhou Limin, Liu Shunqing, Chen Qianqian, Xiao Xiaohu, Wu Jinyan (2023). Diversity and structure of the root-associated bacterial microbiomes of four mangrove tree species, revealed by high-throughput sequencing.. PeerJ.

[B12966219] Tang Lu, Shi Yimeng, Zhang Yilu, Yang Dihe, Guo Changhong (2023). Effects of plant-growth-promoting rhizobacteria on soil bacterial community, soil physicochemical properties, and soil enzyme activities in the rhizosphere of alfalfa under field conditions. Diversity.

[B13595237] Tang Yuanmou, Chen Xiaodie, Hou Liming, He Jing, Sha Ajia, Zou Liang, Peng Lianxin, Li Qiang (2024). Effects of uranium mining on the rhizospheric bacterial communities of three local plants on the Qinghai-Tibet Plateau. Environmental Science and Pollution Research.

[B12966243] Tang Yuanjiang, Zhou Sixuan, Xiao Yuanpin, Zhang Tao, Tao Xiaoyan, Shi Kaizhi, Lu Yuxi, Yang Yueqian, Zhao Yu, Zhao Tian (2024). Exploring the microbial ecosystem of Berchemia
polyphylla
var.
leioclada: a comprehensive analysis of endophytes and rhizospheric soil microorganisms. Frontiers in Microbiology.

[B12964879] Wan dan, Zhou Huoming, Hu Yue, Yu jiang, Gan Guoquan (2022). Plant community structure and species diversity in ecological restoration area of water-level-fluctuating zone in Wudongde reservoir. Ecology and Environmental Monitoring of Three Gorges.

[B12966060] Wang Junsong, Fu Wenjiang, Sun Chenyu, Cai Shuai, Tang Cheng (2022). *Funneliformis
mosseae* inoculation enhances *Cucurbita
pepo* L. plant growth and fruit yield by reshaping rhizosphere microbial community structure. Diversity.

[B13393934] Wang Ziting, Li Tong, Wen Xiaoxia, Liu Yang, Han Juan, Liao Yuncheng, DeBruyn Jennifer M (2017). Fungal communities in rhizosphere soil under conservation tillage shift in response to plant growth.. Frontiers in Microbiology.

[B13595174] Wu Liujie, Wu Weijun, Mao Lixia, Wang Yongzhuang, Liu Di, An Fengxuan, Liang Junrong, Wu Danmiao, Ye Jieping, Wei Xiulan, Li Yongzhu (2025). Rhizosphere microbial diversity and functional roles in tea cultivars: insights from high-throughput sequencing and functional isolates. Plant Signaling & Behavior.

[B13380918] Wu Qiong, Li Qiuhua, Luo Huan, Chen Qian, Chen Huaxiang, Dong Yanjun, Li Shenghua (2022). Comparison in phytoplankton diversity-productivity-community stability between river-type reservoir and lake-type reservoir. Journal of Oceanology and Limnology.

[B13386103] Xiong Wu, Li Rong, Ren Yi, Liu Chen, Zhao Qingyun, Wu Huasong, Jousset Alexandre, Shen Qirong (2017). Distinct roles for soil fungal and bacterial communities associated with the suppression of vanilla *Fusarium* wilt disease. Soil Biology and Biochemistry.

[B13389032] Xu Daolong, Yu Xiaowen, Yang Junbo, Zhao Xupeng, Bao Yuying (2020). High-throughput sequencing reveals the diversity and community structure in rhizosphere soils of three endangered пlants in Western Ordos, China.. Current microbiology.

[B12964889] Yin Jun, Yuan Zhe, Yan Denghua, Yang Zhiyong, Wang Yongqiang (2018). Addressing climate change impacts on streamflow in the Jinsha River Basin based on CMIP5 climate models. Water.

[B13381065] Yi Xuemei, Huang Yuanyang, Xing Qiao, Chen Qiao, Wu Shengjun (2024). *Cynodon
dactylon* and sediment interaction in the three gorges reservoir: Insights from a three-year study. Land.

[B12966071] Yu Chengfeng, Cao Jicheng, Du Wen, Zhu Zhiyong, Xu Min (2022). Changes in the population and functional profile of bacteria and fungi in the rhizosphere of *Suaeda
salsa* is driven by invasion of *Spartina
alterniflora*. Ecological Indicators.

[B12966155] Zhang Tao, Wang Zhongke, Lv Xinhua, Li Yang, Zhuang Li (2019). High-throughput sequencing reveals the diversity and community structure of rhizosphere fungi of *Ferula
sinkiangensis* at different soil depths.. Scientific Reports.

[B12966175] Zhang Xuejiang, Wang Heyun, Que Yawei, Yu Dazhao, Wang Hua (2021). The influence of rhizosphere soil fungal diversity and complex community structure on wheat root rot disease.. PeerJ.

[B13393258] Zhao Yuxiang, Lou Yicheng, Qin Weizhen, Cai Jingjie, Zhang Pan, Hu Baolan (2022). Interval aeration improves degradation and humification by enhancing microbial interactions in the composting process.. Bioresource Technology.

[B12964796] Zhou Lanfang, Wu Shengjun, Ma Maohua (2023). First insights into diversity and potential metabolic pathways of bacterial and fungal communities in the rhizosphere of *Argemone
mexicana* L. (Papaveraceae) from the water-level-fluctuation zone of Wudongde Reservoir of the upper Yangtze river, China. Biodiversity Data Journal.

[B12964823] Zhou Lanfang, Wu Shengjun, Ma Maohua, Zou Hang, Huang Jinxia, Yang Jun (2024). Rhizosphere microbial community structure in the water-level-fluctuation zone under distinct waterlogging stresses. One Ecosystem.

[B12964940] Zhou Yingjie, Tang Yanni, Hu Chengxiao, Zhan Ting, Zhang Simin, Cai Miaomiao, Zhao Xiaohu (2021). Soil applied Ca, Mg and B altered phyllosphere and rhizosphere bacterial microbiome and reduced Huanglongbing incidence in Gannan Navel Orange. Science of The Total Environment.

