# Supplementary material for: Comparative analysis on rhizosphere microorganisms of Cynodon dactylon (Poaceae) from the natural restoration area and eco-restoration engineering area in water-level-fluctuation zone of Jinsha River-type reservoirs, China
Source: Biodivers Data J. 2025 Dec 30;13:e159524. doi: 10.3897/BDJ.13.e159524 (PMC12776026; doi:10.3897/BDJ.13.e159524)
Supplement: Supplementary material 1 — Supplementary file [file bdj-13-e159524-s001.docx]

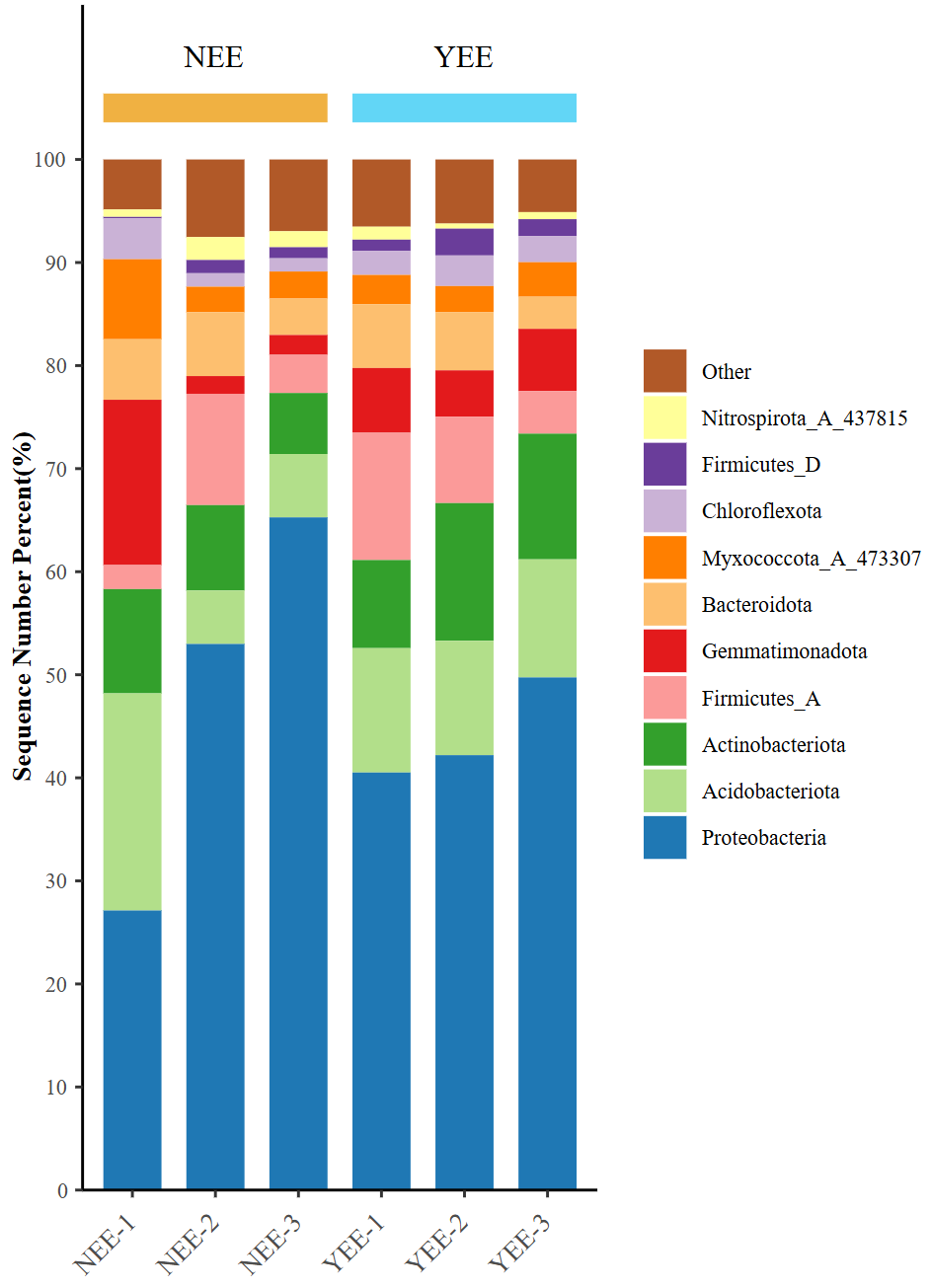


**Fig. S1.** Composition variation of rhizosphere bacteria in *C. dactylon* betweem NEE/ YEE samples at the phylum level.


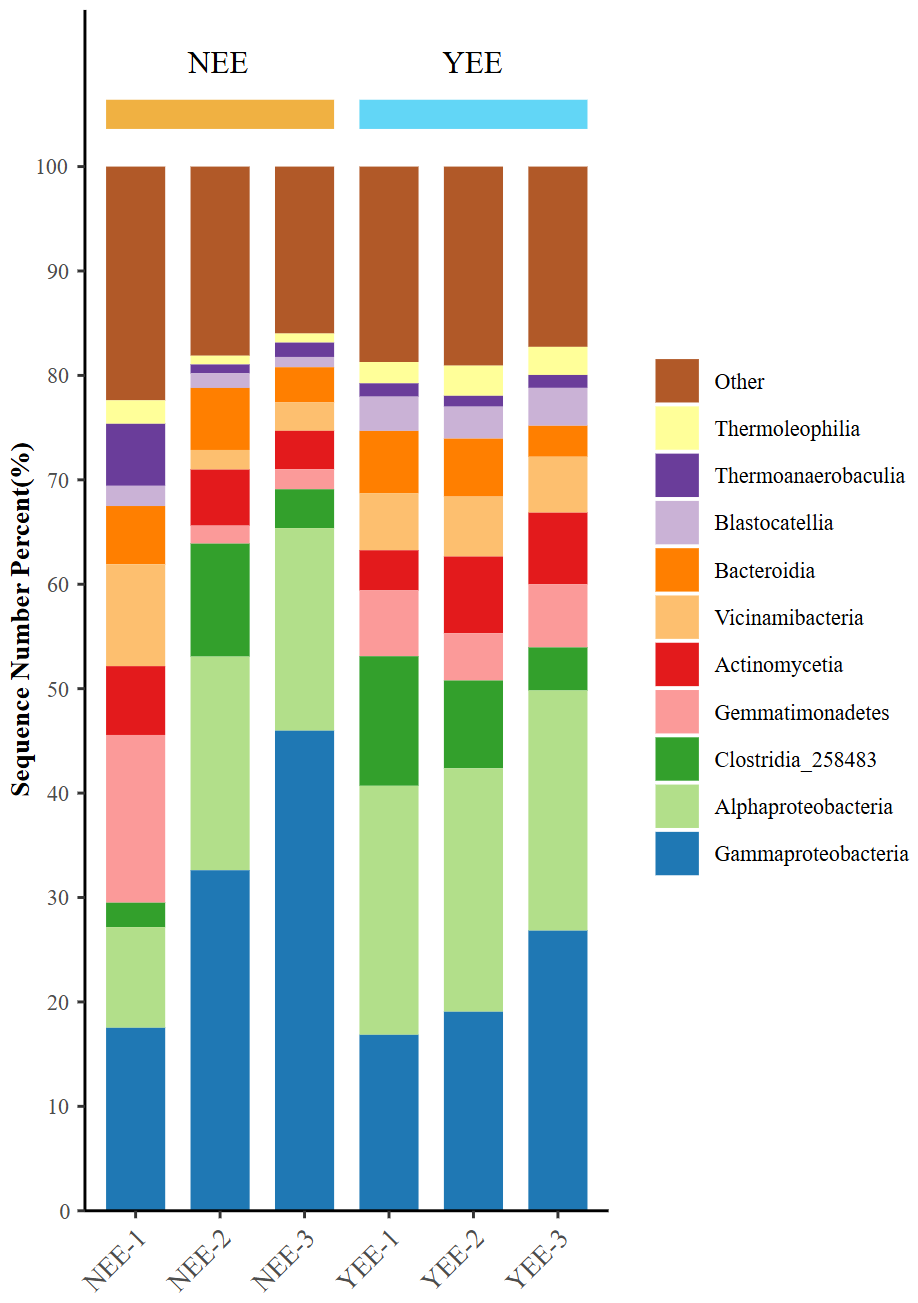


**Fig. S2.** Composition variation of rhizosphere bacteria in *C. dactylon* betweem NEE/ YEE samples at the class level.


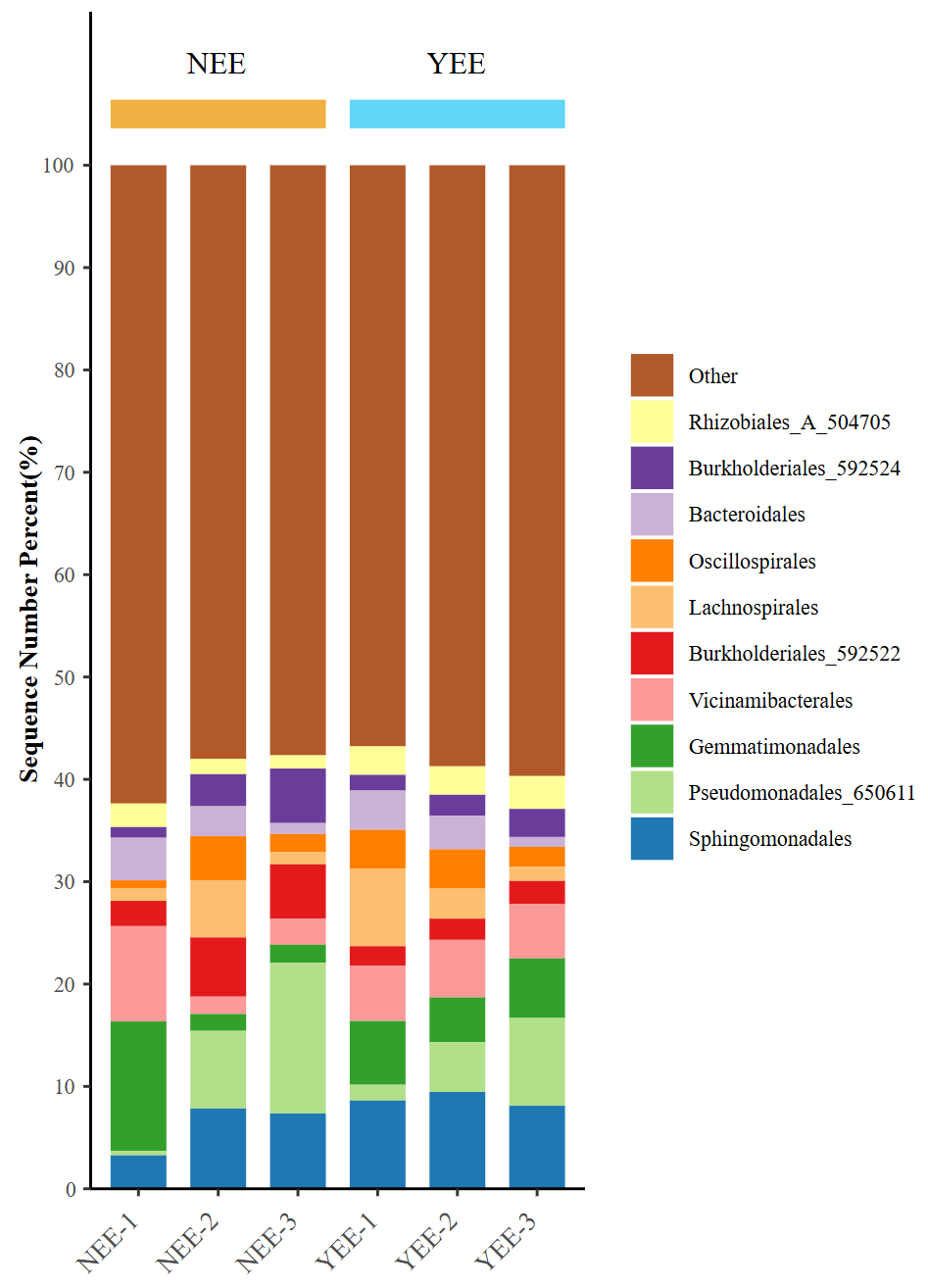


**Fig. S3.** Composition variation of rhizosphere bacteria in *C. dactylon* betweem NEE/ YEE samples at the order level.


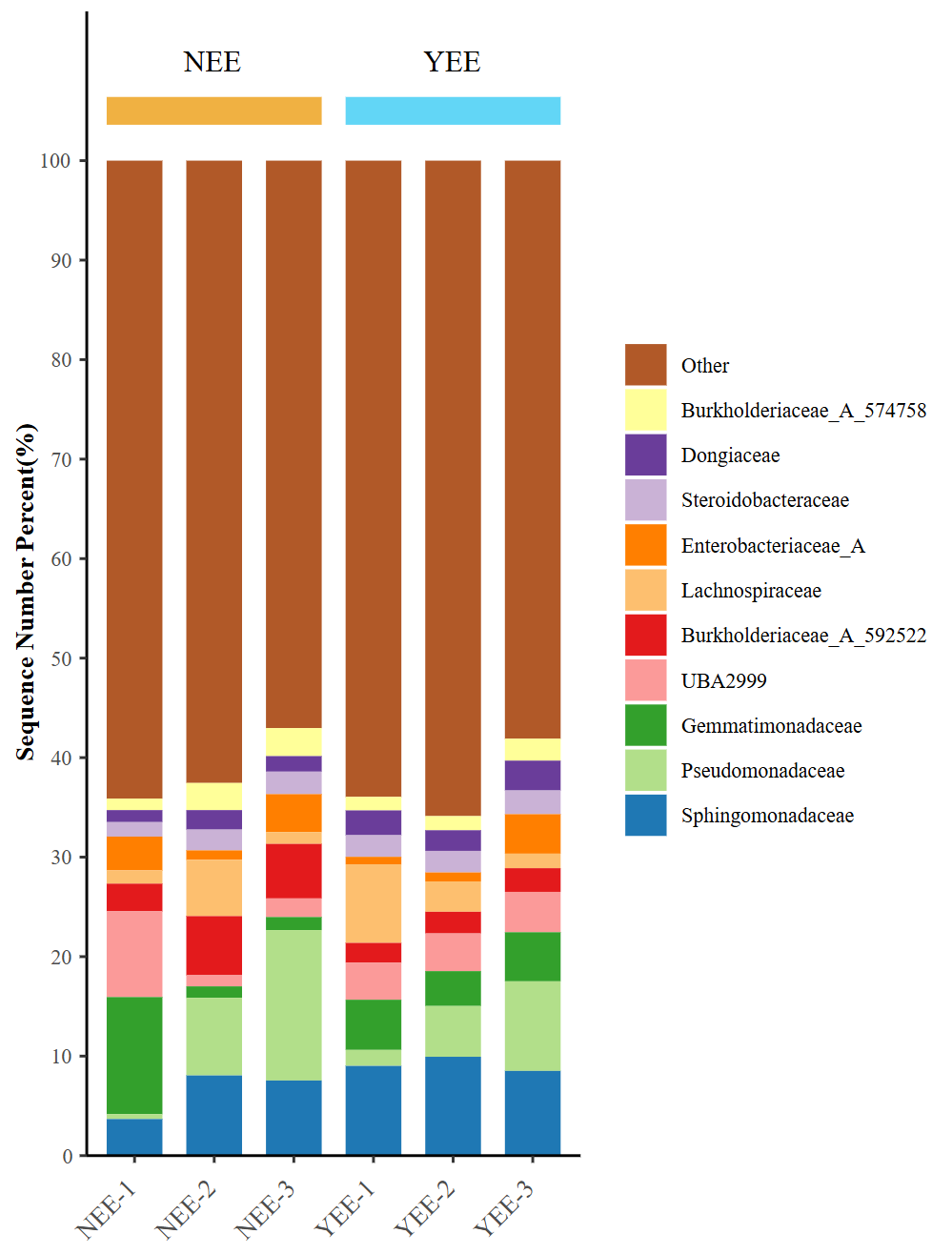


**Fig. S4.** Composition variation of rhizosphere bacteria in *C. dactylon* betweem NEE/ YEE samples at the family level. UBA2999 belongs to *Vicinamibacterales*.


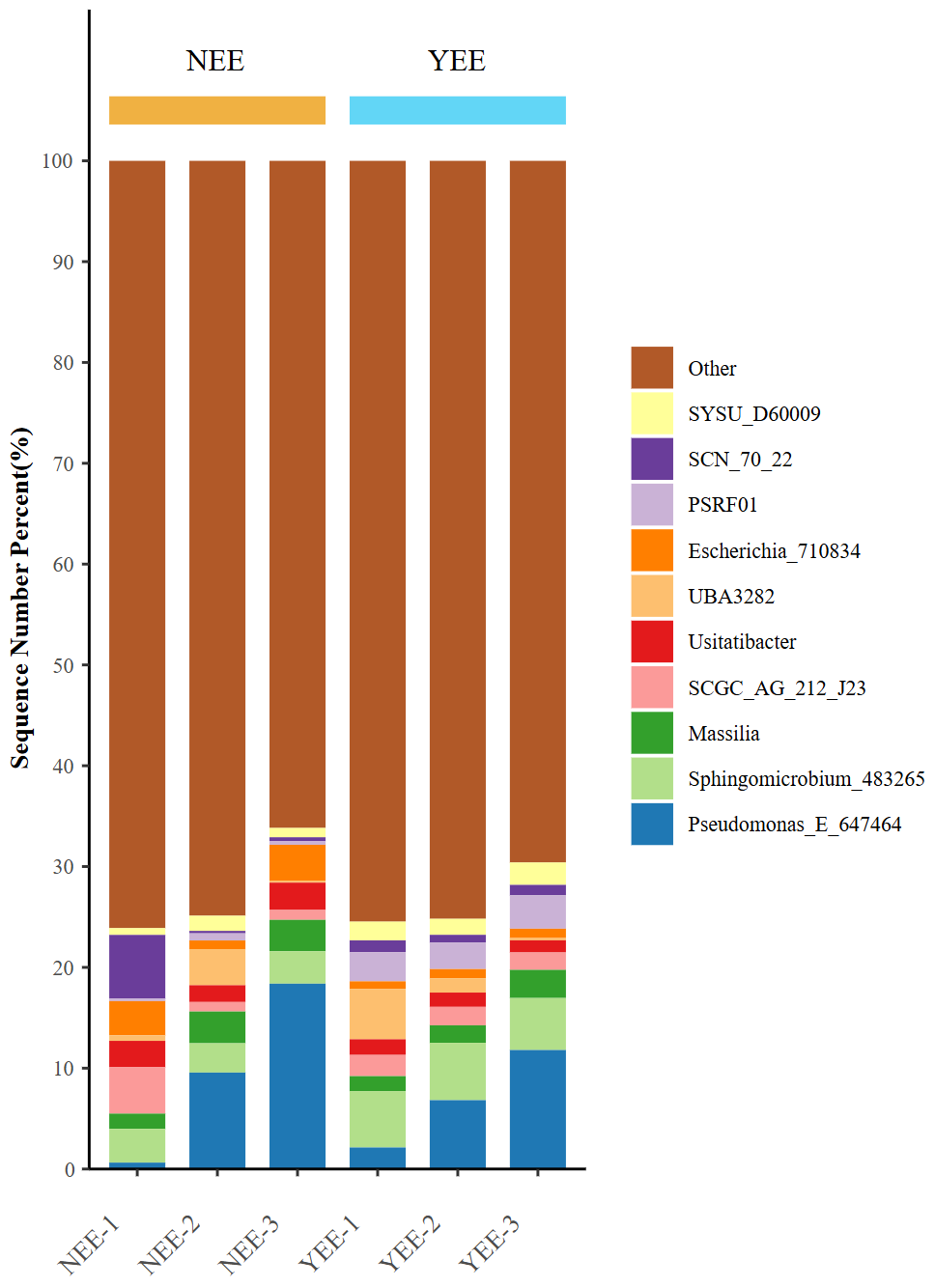


**Fig. S5.** Composition variation of rhizosphere bacteria in *C. dactylon* betweem NEE/ YEE samples at the genus level. SYSU_D60009 belongs to *Dongiaceae*; SCN_70_22 belongs to *Gemmatimonadaceae*; PSRF01 belongs to *Pyrinomonadaceae*; UBA3282 belongs to *Lachnospiraceae*; SCGC_AG_212_J23 belongs to *Gammaproteobacteria*.


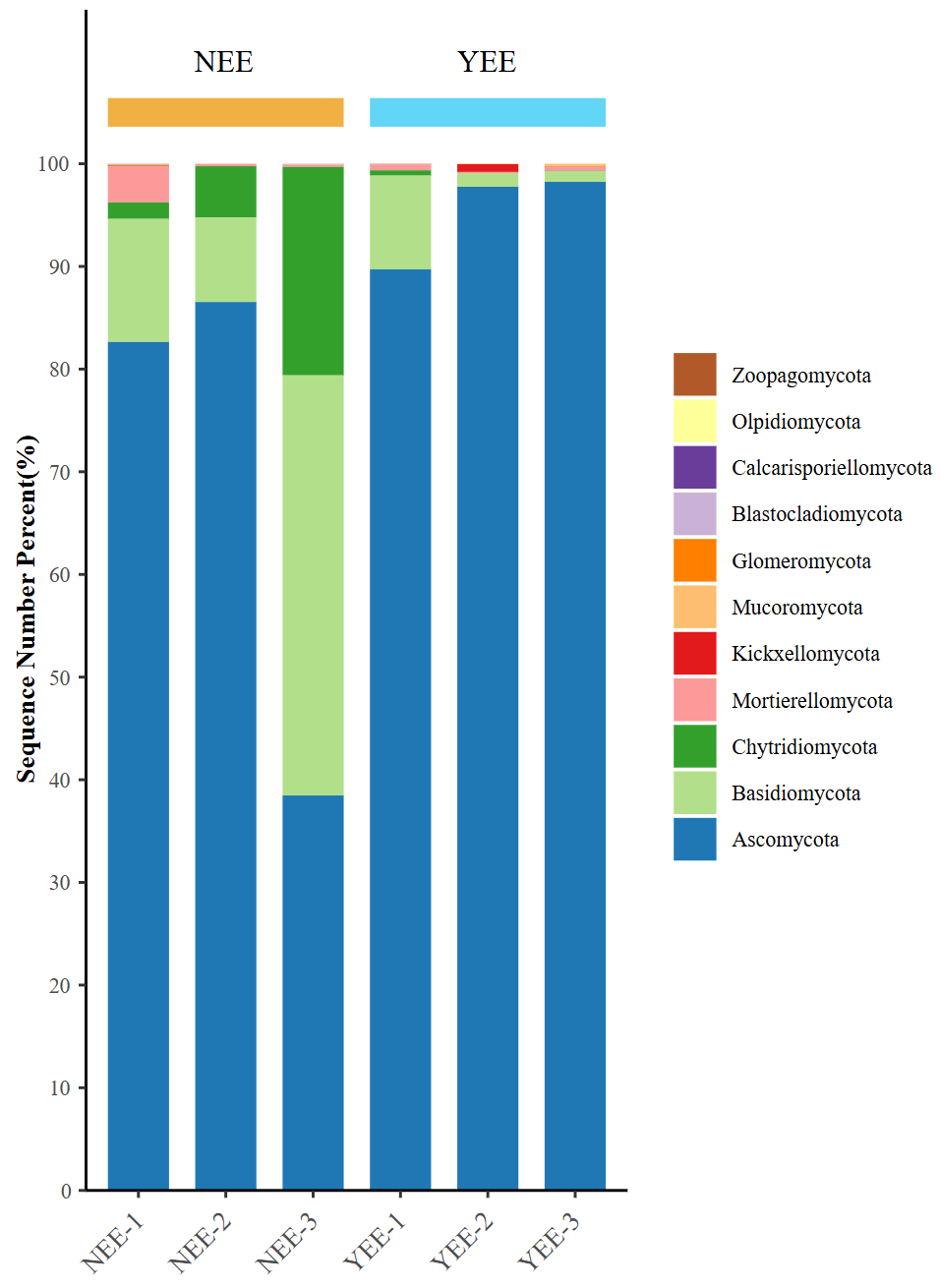


**Fig. S6.** Composition variation of rhizosphere fungi in *C. dactylon* betweem NEE/ YEE samples at the phylum level.


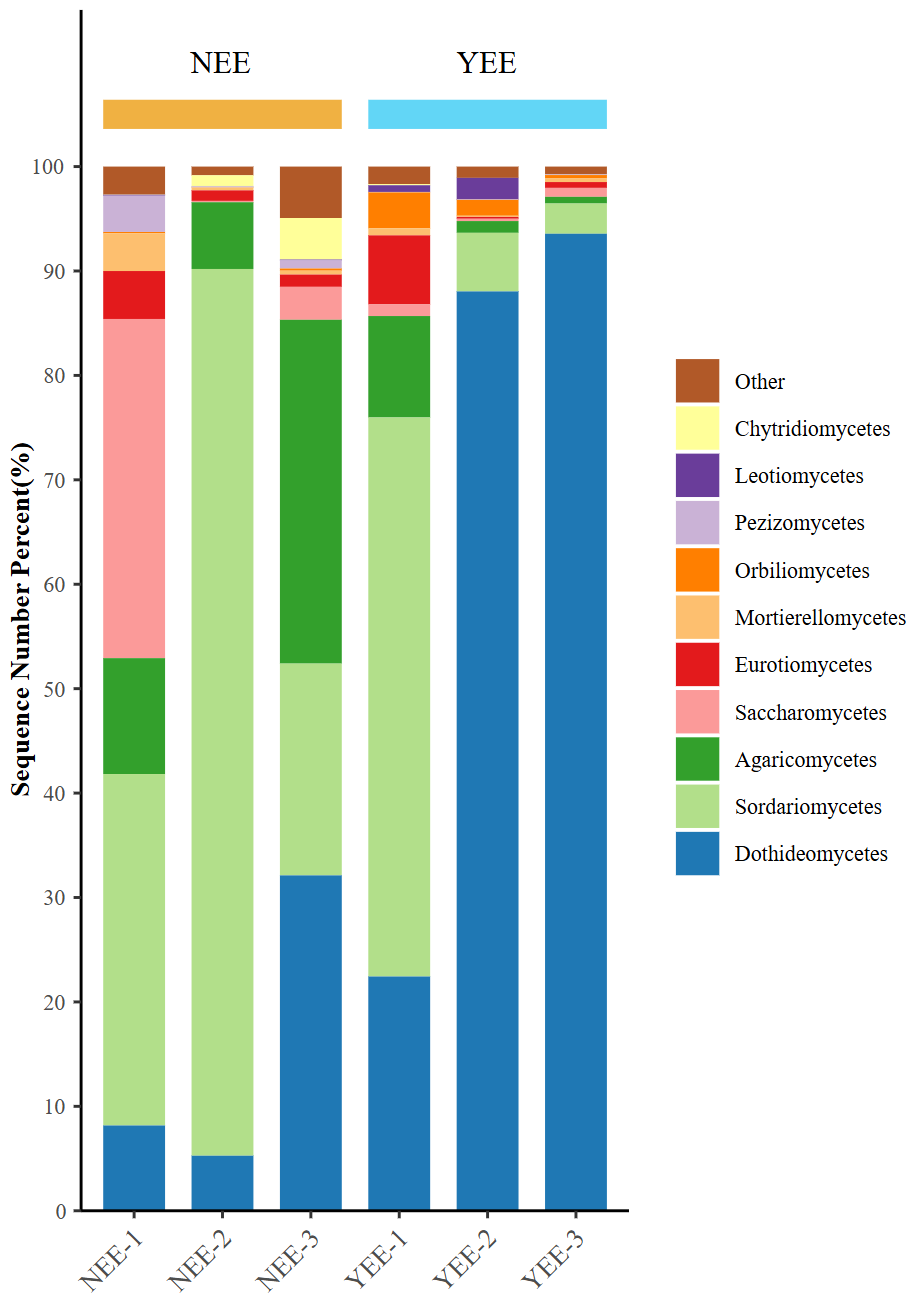


**Fig. S7.** Composition variation of rhizosphere fungi in *C. dactylon* betweem NEE/ YEE samples at the class level.


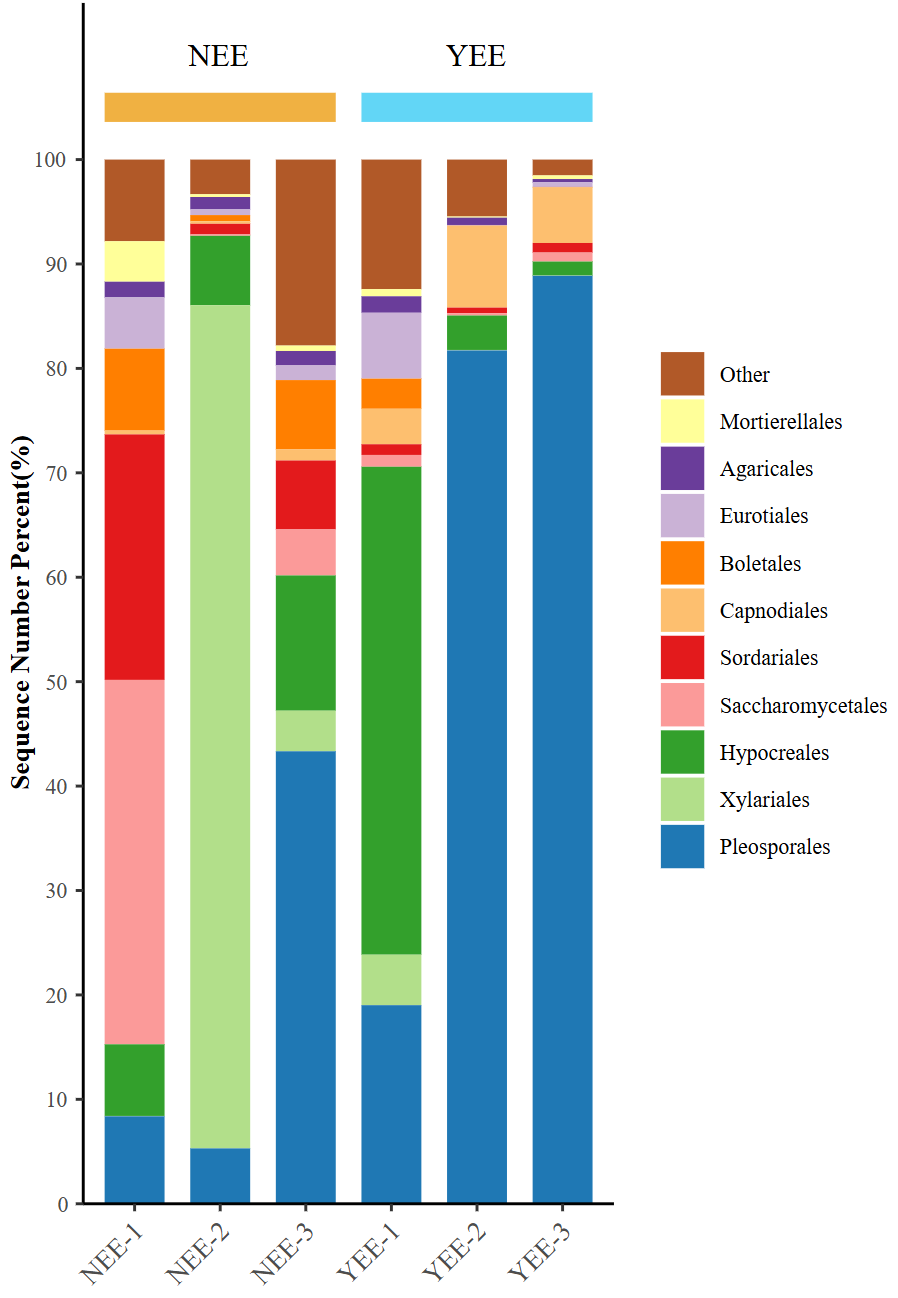


**Fig. S8.** Composition variation of rhizosphere fungi in *C. dactylon* betweem NEE/ YEE samples at the order level.


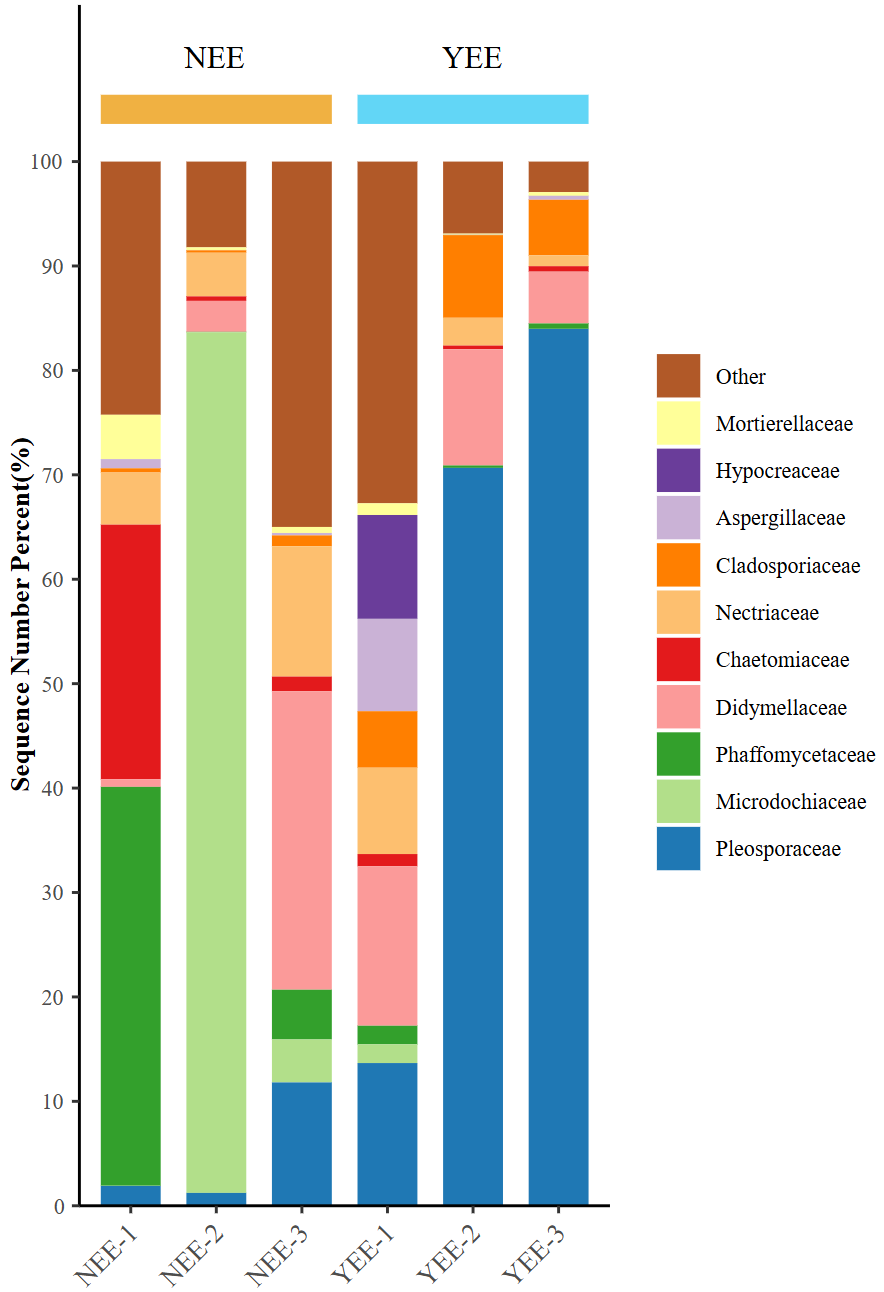


**Fig. S9.** Composition variation of rhizosphere fungi in *C. dactylon* betweem NEE/ YEE samples at the family level.


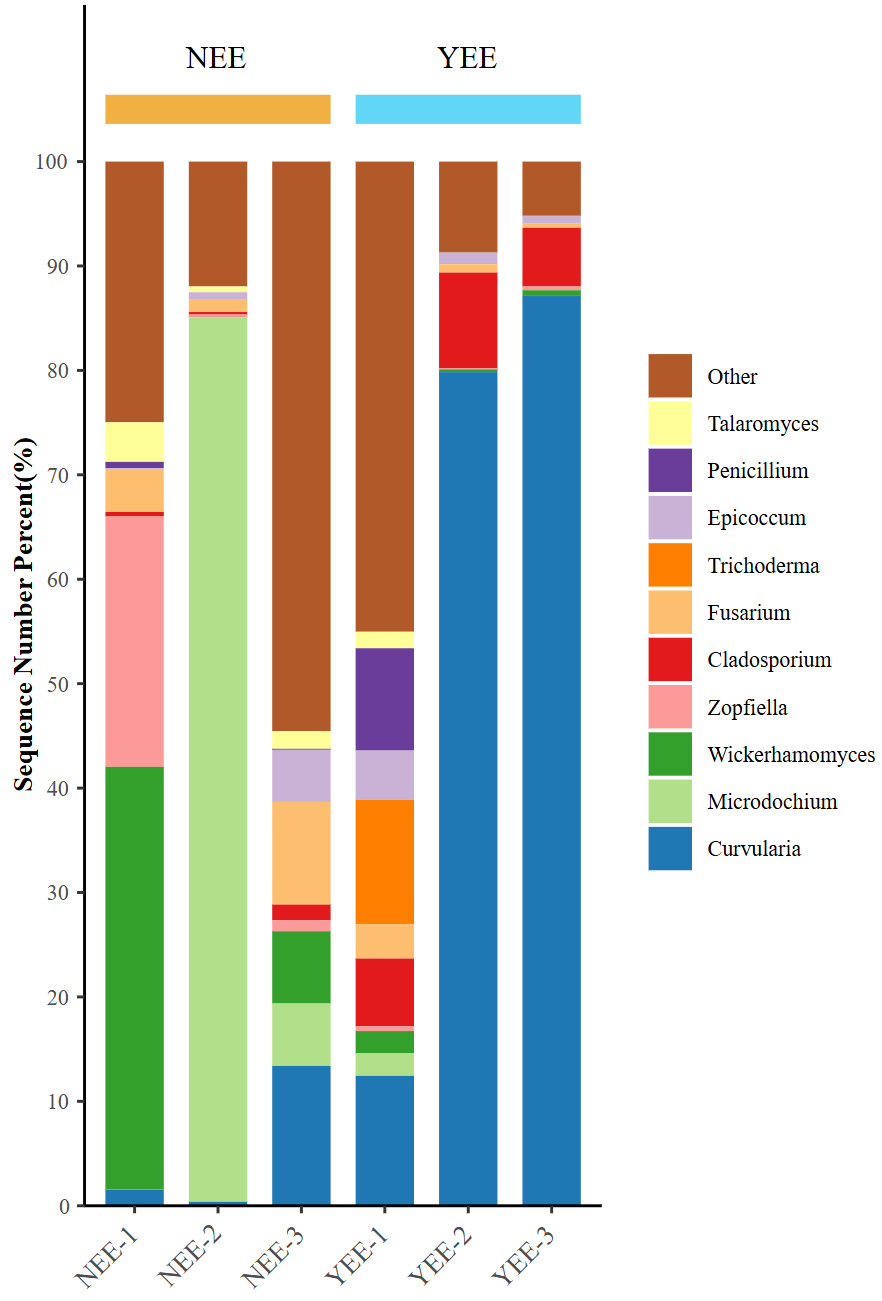


**Fig. S10.** Composition variation of rhizosphere fungi in *C. dactylon* betweem NEE/ YEE samples at the genus level.


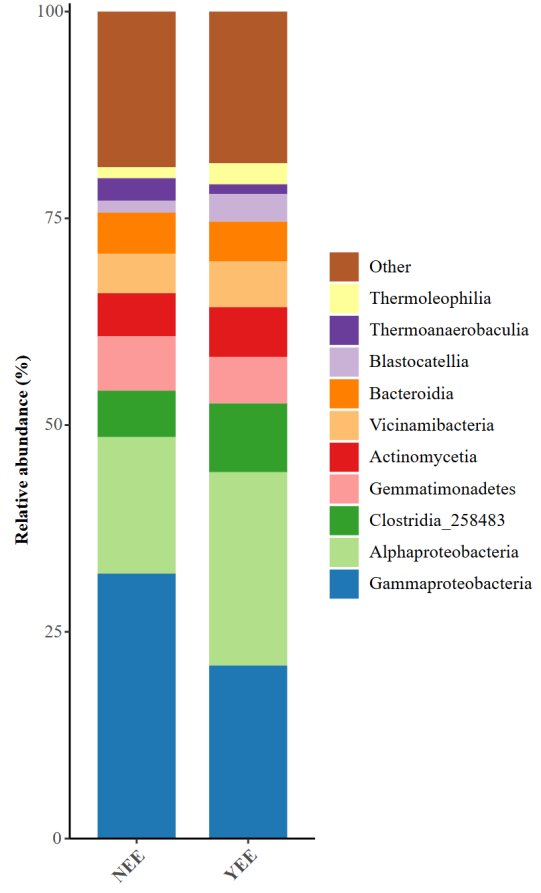


**Fig. S11.** Composition of rhizosphere bacteria in *C. dactylon* of NEE and YEE at the class level.


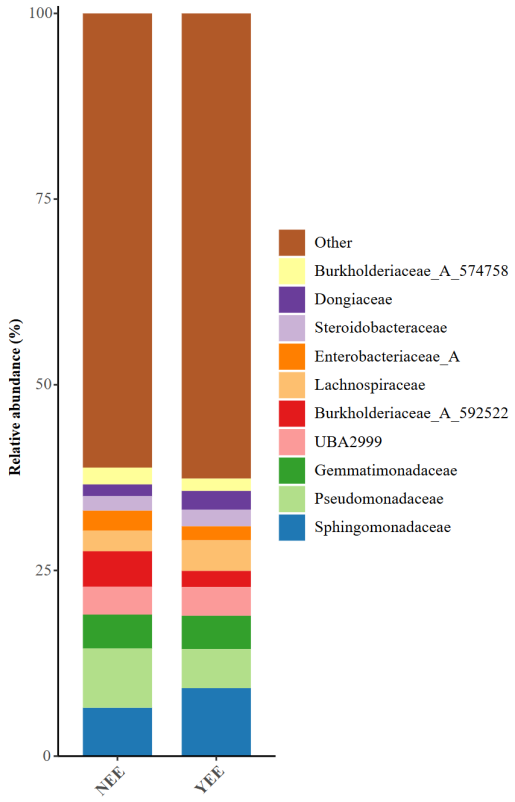


**Fig. S12.** Composition of rhizosphere bacteria in *C. dactylon* of NEE and YEE at the family level.


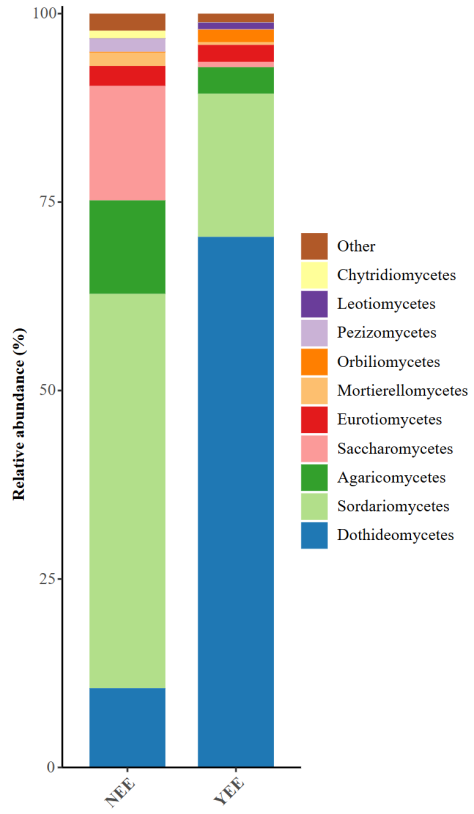


**Fig. S13.** Composition of rhizosphere fungi in *C. dactylon* of NEE and YEE at the class level.


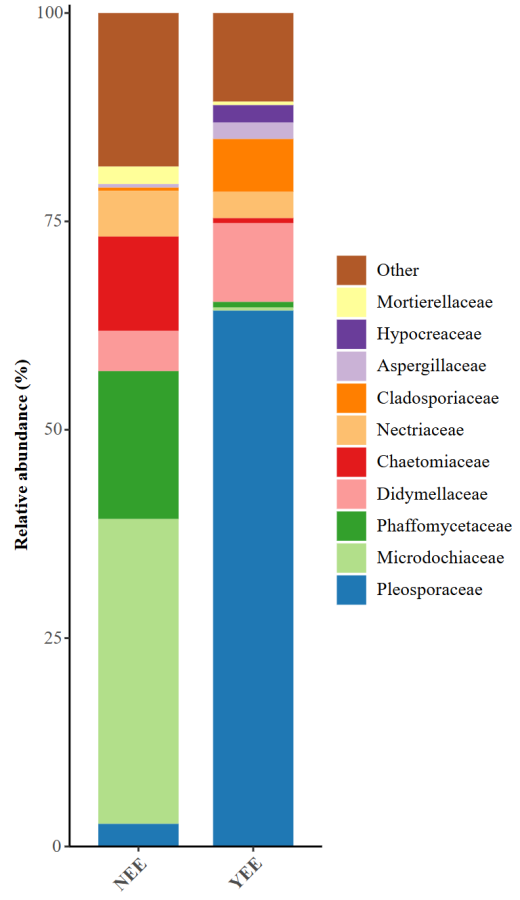


**Fig. S14.** Composition of rhizosphere fungi in *C. dactylon* of NEE and YEE at the family level.
